# Malting barley carbon dots-mediated oxidative stress promotes insulin resistance in mice via NF-κB pathway and MAPK cascade

**DOI:** 10.1186/s12951-022-01543-1

**Published:** 2022-07-16

**Authors:** Boya Zhang, Lidong Yu, Ruijiao Zhu, Xiangjuan Wei, Xingpei Fan, Hailong Hu, Daqian Yang, Haining Du, Meimei Zhao, Li Li, Yuri Oh, Yujie Feng, Ning Gu

**Affiliations:** 1 School of Life Science and Technology, Harbin Institute of Technology, Harbin, 150001 China; 2 State Key Laboratory of Urban Water Resource and Environment, Harbin Institute of Technology, Harbin, 150006 China; 3 School of Physics, Harbin Institute of Technology, Harbin, 150001 China; 4 Department of Medicine, Renal Electrolyte and Hypertension Division, Department of Genetics, Perelman School of Medicine, University of Pennsylvania, Philadelphia, PA 19019 USA; 5 Faculty of Education, Wakayama University, Wakayama, Japan

**Keywords:** Carbon dots, Oxidative stress, Inflammatory responses, MAPK cascade, Insulin resistance, Hyperglycemia

## Abstract

**Background:**

Food-borne carbon dots (CDs) are widely generated during food processing and are inevitably ingested by humans causing toxicity. However, the toxic effects of food-borne CDs on the blood glucose metabolism are unknown.

**Results:**

In this study, we brewed beer via a representative strategy and extracted the melting-barley CDs (MBCDs) to explore the toxic effects on blood glucose in mice. We found the accumulation of fluorescent labeled MBCDs in various organs and oral administration of MBCDs can cause visceral toxicity, manifested as liver damage. Mice were orally administered MBCDs (5 and 25 mg/kg) for 16 weeks, and increased levels of fasting blood glucose were observed in both MBCDs-treated groups. Transcriptomic analyses revealed that MBCDs activate oxidative stress, inflammatory responses, the MAPK cascade, and PI3K/Akt signaling in mice livers. Mechanistically, MBCDs exposure-induced reactive oxygen species (ROS) overproduction activates the nuclear factor-κB (NF-κB) signaling pathway and MAPK cascade, thereby promoting phosphorylated insulin receptor substrate (IRS)-1 at Ser307 and inducing insulin resistance (IR). Meanwhile, the IR promoted gluconeogenesis, which enhanced MBCDs-induced hyperglycemia of mice. Importantly, inhibition of the ROS significantly attenuated the MBCDs-induced inflammatory response and MAPK cascade, thereby alleviating IR and hyperglycemia in mice.

**Conclusion:**

In summary, this study revealed that MBCDs promote ROS overproduction and thus induced IR, resulting in imbalance of glucose homeostasis in mice. More importantly, this study was further assessed to reveal an imperative emphasis on the reevaluation of dietary and environmental CDs exposure, and has important implications for T2DM prevention research.

**Graphical Abstract:**

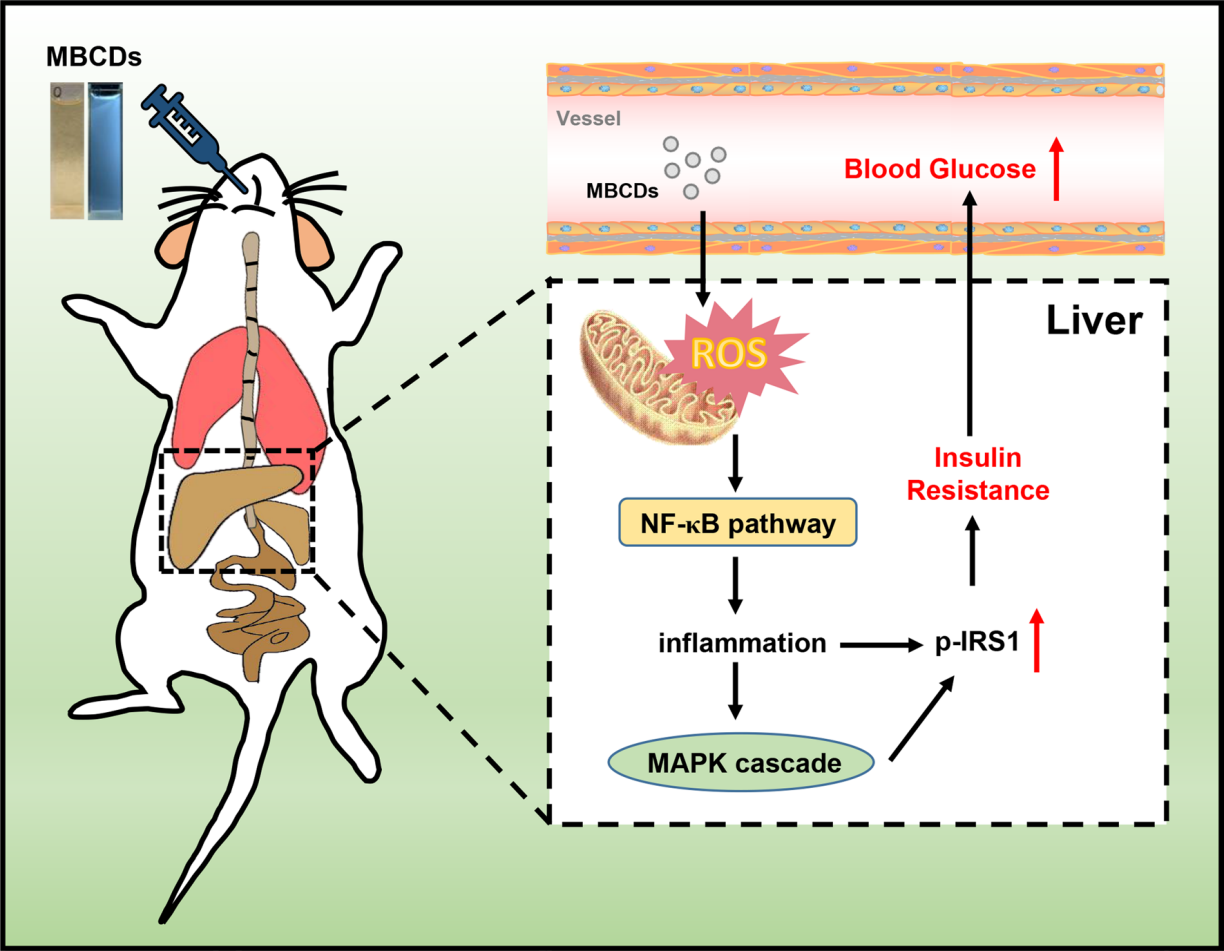

**Supplementary Information:**

The online version contains supplementary material available at 10.1186/s12951-022-01543-1.

## Background

As emerging carbon-based nanoparticles, carbon dots (CDs) have received extensive research attention owing to their small size (below 10 nm), large specific surface area, and fluorescent properties [1]. Previous studies on CDs have focused on their preparation and application while ignoring the potential health risks. Due to the carbonization process of the organic ingredients, food-borne CDs can be generated when heated [2]. They are then inevitably ingested by humans because they are present in daily food items such as coffee [3], baked food [4], bakery products [5, 6], roasted meat [7], pizza [8], and especially, beer [9, 10], the most consumed alcoholic beverage worldwide. Over the past 50 years, total global beer consumption has risen sharply in emerging economies [11, 12]. Long-term, excessive beer consumption has been associated with metabolic disease outcomes (obesity, diabetes and non-alcoholic fatty liver disease) and causes preventable death [13, 14]. The correlation between beer and the inevitable intake of CDs in beer is worth exploring.

Type II diabetes mellitus (T2DM) is a chronic endocrine disease that is prone to multiple complications and poses a serious social and economic burden [15]. Genetic background, dietary changes, and environment have contributed to this disease’s prevalence. The liver, a vital metabolic organ, plays a crucial role in metabolic disease and is an important target organ of insulin to control hepatic glucose homeostasis [16]. As a hypoglycemic hormone, insulin mediates its biological effects via insulin receptors in target cells, which promote glucose uptake and inhibit liver gluconeogenesis to maintain glucose homeostasis [17]. Insulin resistance (IR), the core defect of T2DM, is a pathological state involving the inability of receptors to react efficiently in response to insulin stimulation, resulting in an intracellular glucose metabolism disorder [18]. Gluconeogenesis is also a major driving force for hyperglycemia under IR conditions by promoting endogenous glucose production [19]. Currently, the prevalence of T2DM caused by a diet-induced imbalance of glucose homeostasis is increasing yearly, and we cannot ignore the impact of food nanostructure contamination on blood glucose.

Reactive oxygen species (ROS), the byproducts of oxygen metabolism, play a critical role in the toxicological impacts induced by nanoparticles between 1 and 100 nm in size. Persuasive evidence has proved that exposure to carbon-based materials (carbon nanotubes, graphene, and graphene quantum dots) can induce ROS generation, resulting in cytotoxicity to a variety of cell types [20, 21]. Graphene quantum dots induce ROS production, apoptosis, autophagy, and inflammatory response in THP-1 activated macrophages through the p38MAPK and NF-kB mediated signaling pathways [22]. CD-induced ROS production activates Akt/mTOR signaling and increases glutamine metabolism, thus promoting uveal melanoma cell tumorigenicity [23]. However, the toxic impacts of CDs on the endocrine system are not well characterized. Excessive production of ROS in the body can affect cellular mechanisms such as oxidative stress, endoplasmic reticulum (ER) stress, and inflammation, which worsen IR leading to glucose homeostasis imbalance [24]. Previous studies have demonstrated that a series of nano-oxides (TiO_2_, SiO_2_, and ZnO) increased ROS production in mice [25–27]. The excessive ROS activated the MAPK and NF-κB pathways to induce inflammatory responses, which phosphorylated insulin receptor substrate 1 (IRS1) at Ser-307, leading to IR in mice. The nano-plastic particles (80 ± 6 nm) induced overproduction of ROS and activated the PI3K/Akt pathway, which led to IR and was reflected in a glucose homeostasis imbalance in mice [28]. Considering that CDs sized below 10 nm may enter cells easier and produce toxicity, we hypothesized that beer-borne CDs would disrupt glucose homeostasis more severely. Therefore, we investigated the impact of beer-borne CDs on glucose homeostasis imbalance, and the relationship with ROS.

The brewing of beer is a fundamental process that has not changed in millennia, including kilning, gelatinization, boiling, and fermentation of malting barley [29]. However, it is a fact that carbon-based nanoparticles are produced during beer brewing, and may pose a potential health hazard. In this work, we used malting barley to brew beer via a representative strategy and then extracted the CDs for further study. Here, we performed experiments to investigate the disruptive effects of oral intake of malting barley-borne CDs (MBCDs) on blood glucose homeostasis in different dose-groups of mice. Furthermore, we used RNA-seq to detect whole transcriptomes of mouse liver and investigated the pathogenic mechanisms of MBCDs-induced oxidative stress on glucose metabolism disorders in mice.

## Methods

### Preparation of MBCDs

The malting barley, hops and yeast were purchased from a local market (Harbin, China). The brief protocol for brewing beer refers to C.W. Bamforth’s work [29], and the process was as follows: (i) milling of malted grains and mashing: malting barley was mashed in a mill and mixed with warm water. The mash was heated to 73–80 °C for starch gelatinization; (ii) clarification: The clarification of extracted solution was operated at 90–100 °C for 3 h to filter out the insoluble part, and the remaining extract was transferred to a boiling container; (iii) boiling: add hops to the wort and boil for 1–3 h; (iv) fermentation: cool down the wort, supply oxygen, add yeast and ferment for 14 days (6–25 °C), then the self-brewing beer could be obtained. Afterwards, we used a vacuum rotary evaporator to concentrate self-brewing beer and D101 macroporous resin column to further purify it; the fluorescent components were collected and lyophilized to obtain MBCDs. The MBCDs were stored in a dark chamber at 4 °C for further characterization.

### Materials characterization

The surface morphology of the MBCDs was examined by transmission electron microscopy (TEM, FEI Tecnai 30) and Atomic Force Microscope (AFM, Dimension Fastscan. The phase composition of the MBCDs was characterized by X-ray diffraction (Rigaku D/max 2550 VB + 18 kW with Cu Kα′ radiation) and X-ray photoelectron spectroscopy (PHI Quantera XPS). The Fourier transform infrared (FTIR) spectra of the MBCDs was collected by the Thermo Nicolet iS50 FT-IR instrument (Thermo Scientific, USA). The fluorescence properties were determined by fluorimeter (FluoroMax-4, HORIBA Jobin Yvon, France).

### Animals and treatment

All animal studies were approved by the Ethics Research Committee of the School of Life Science and Technology, Harbin Institute of Technology, and carried out in accordance with the guidelines for the care and use of experimental animals approved by the Heilongjiang Province People’s Congress. The ethical number of our study is IACUC-2021002.

Twenty-four CD-1 (ICR) mice (21.95 ± 0.29 g) were purchased from Harbin Medical University and were cohoused for a week in our animal room. The mice were housed in temperature-controlled cages (21–24 ℃, 50% ± 60% relative humidity) and with a light cycle (12 h light/dark). The mice were given a CHOW diet (KeaoCo., Ltd., Beijing, China) and autoclaved water. Divide mice into random three groups (n = 8) for further experimentation: control (C), 5 mg/kg (L), 25 mg/kg (H). A solution of MBCDs was prepared daily in an ultrapure sterile and ultrasonicated for 10 min. The solution was then orally administered to the mice for 16 weeks while the control group was given an equal volume of sterile water. The fasting body weight and blood glucose of mice were measured every two weeks. At week 16, mice fasted for 16 h were anesthetized by intraperitoneal injection of barbiturates, and subsequently executed. The heart blood was collected and centrifuged at 3000 rpm for 30 min and finally stored at – 20 ℃. The tissues were extracted, weighed, and then stored at – 80 ℃.

### Biodistribution of CY7-labeled CDs

To analyze the absorption of MBCDs, mice were given 200 μL of CY7-labeled MBCDs (CY7: 1 mg/mL, MBCDs: 300 mg/mL) by intravenous tail injections to evaluate their biodistribution. The C group was orally administered an equal volume 0.9% NaCl solution. After 0.5 h, the mice were sacrificed and major organs were taken to be observed and imaged on a multifunctional in vivo imaging system (Molecular Devices, San Jose, CA).

### Blood collection and analysis

Mice were fasted for 16 h every 2 weeks and tail vein blood was collected. Measurement of blood glucose levels in mice tail vein blood using a glucose assay kit (Wako Pure Chemical Industries, Ltd., Osaka, Japan).

### Glucose and insulin tolerance test

At week 14, an OGTT experiment was performed to examine glucose tolerance in mice. After fasting for 16 h, mice were orally administered glucose solution (1.5 g/kg), and tail vein blood was collected at 0, 30, 60 and 120 min for glucose and insulin level measurement. The blood insulin was measured using a mouse insulin ELISA kit (Shibayagi Co., Ltd., Gunma, Japan). At week 15, an ITT experiment was performed to examine insulin sensitivity in mice. After fasting for 16 h, mice were intraperitoneal injected with insulin (0.4 IU/kg), and tail vein blood was collected at 0, 30, 60 and 120 min for glucose level measurement.

### Histology

The tissues were fixed in 10% formalin for 24 h and then embedded in paraffin. The 5-μm-thick sections of liver, pancreas, kidney, and small intestine were stained with hematoxylin (H&E) for the histomorphological observations. A TUNEL kit (Absin, Shanghai, China) was used to detect the apoptosis degree of liver cells and operation schemes in accordance with the manufacturer’s protocols. The images were captured by a macro zoom fluorescence microscope (Axio Zoom.V16, Agilent, USA).

### Detection of biomarkers

We placed a piece of liver tissue into 500 μL precooling PBS and it was homogenized on ice and then centrifuged at 12,000 rpm for 10 min to obtain the liver homogenate. The protein concentration was tested by a BCA protein quantitative Kit (Absin, Shanghai, China). The ROS-related indicators and liver function biomarkers in serum and liver homogenate were measured using commercial kits (Nanjing Jiancheng, China), including total superoxide dismutase (T-SOD), glutathione (GSH), malondialdehyde (MDA), aspartate transaminase (AST), and alanine aminotransferase (ALT). All biomarkers were detected in accordance with the manufacturer’s protocols.

### RNA sequencing and data analysis

In order to analyze the effect of mouse liver total transcriptome after MBCD treatment, 15 mg liver was collected to isolate the total RNA using RNAiso Plus reagent (Takara Biochemicals, USA) according to the manufacture’s protocol. Three livers of C and H group were sequenced, and the data were analyzed. The RNA sequencing was performed at Wuhan MetWare Biotechnology Co., Ltd. (www.metware.cn) using Illumina Genome Analyzer II system (Illumina). Functional annotation analysis of differential genes was performed using the Annotation, Visualization and Integrated Discovery, (DAVID, https://david.ncifcrf.gov/home.jsp).

### Quantitative real-time polymerase chain reaction (qRT–PCR) analysis

Liver tissues were collected to isolate the total RNA using RNAiso Plus reagent (Takara Biochemicals, USA) according to the manufacture's protocol. The liver RNA concentrations and purity were assessed and checked using the NanoDrop 2000 spectrophotomer (Thermo Scientific, USA). Complementary DNA (cDNA) was synthesized using 1 μg RNA according to the PrimeScript RT reagent Kit (Takara Biotechnology, USA). The expression of mRNA was tested by a ABI 7500a real-time PCR machine (Applied Biosystems, USA) using the SYBR Green system (Takara Biochemicals, USA) and. β-actin was used to normalize the mRNA level. Primer sequences used are listed in Table S1, Supporting Information.

### Western blotting analysis

Total proteins were extracted from mice liver by RIPA Lysis Buffer, and then quantified using the BCA protein kit (Absin, China). Proteins were separated by SDS–PAGE, and transferred to PVDF membranes (Bio-Rad Laboratories, CA, USA). PVDF membranes were blocked in 5% nonfat milk for 1 h, then incubated with antibodies against NRF2, HO-1, phospho-IRS1 (Ser307), phospho-Akt (Ser473), IRS1, Akt, phospho-NFκB-p65, phospho-IκBα, phospho- JNK, phospho-p38-MAPK, NFκB-p65, IκBα, JNK1, p38-MAPK and β-actin for 14–16 h at 4 °C.

### Treatment with antioxidants in mice

Thirty-two six-week-old male mice were randomly divided into four groups: C, MBCDs, MBCDs + Res, and MBCDs + NAC. The CDs group was given 25 mg/kg MBCD solution. A mixture of CDs (25 mg/kg) and resveratrol (100 mg/kg) or N-Acetyl-L-Cysteine (100 mg/kg) were given to the MBCDs + Res or MBCDs + NAC groups. The control group was administered an equal volume of sterile water. During the treatment, tail vein blood was collected after fasting for 16 h every 2 weeks to determine fasting blood glucose. An OGTT test was performed after 14 weeks of treatment to detect glucose homeostasis. After 15 weeks of treatment, the tissues of mice were collected for further analysis.

### Statistical analysis

Data are presented as mean ± SEM. The significance of difference among all groups was tested by one-way analysis of variance (ANOVA) test and the comparison between each two groups was carried out using Student’s *t*-test. The P-values < 0.05 were considered significant. All comparisons were carried out by GraphPad Prism version 5.0.

## Results

### Phase and composition characterization of MBCDs and BCDs

**Fig. 1:**
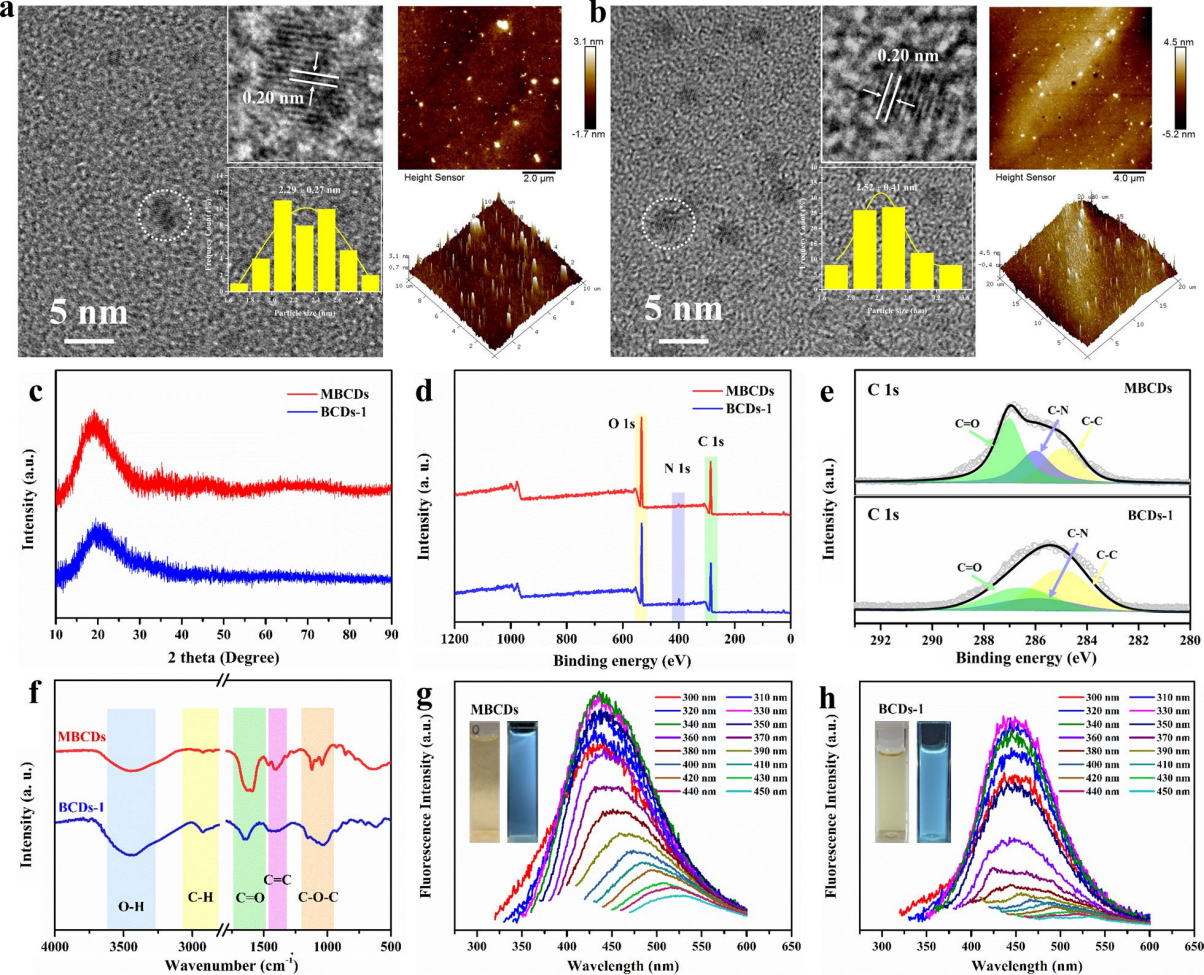
MBCDs characterization. **a**, **b** HRTEM and AFM images of the water-dispersed MBCDs and BCDs-1 (inset: local high-resolution HRTEM images and particle size statistics). **c** XRD spectrum. **d** XPS spectrum and **e** high revolution spectra of C 1 s. **f** FT-IR spectrum. **g**, **h** photoluminescence spectrum excited with different wavelength, and digatal images of solution under daylight and ultraviolet conditions, respectively

In this study, MBCDs and Beer-CDs (BCDs) from self-brewing beer and different commercial beer (1: Snow, 2: Tsingtao, and 3: Budweiser) were extracted by a D101 macroporous resin column for comparative analysis. Transmission electron microscopy (TEM) and atomic force microscopy (AFM) were first performed to characterize the nanostructure of the MBCDs and BCDs-1. As shown in Fig. 1a and b, the TEM images display MBCDs and BCDs-1 are well dispersed with narrow average diameters (2.29 ± 0.27 nm and 2.52 ± 0.41 nm) and exhibit substantial crystalline structures. Moreover, the corresponding high-resolution transmission electron microscopy (HRTEM) images show the same apparent interplanar spacing of 0.20 nm for the MBCDs and BCDs-1, and AFM technology was used to measure the three-dimensional morphological structure of MBCDs and BCDs-1, which exhibit the sharp morphology with an average height of 3.1 nm and 4.5 nm, respectively. In Fig. 1c, the X-ray diffraction (XRD) pattern of MBCDs and BCDs-1 shows that the peak at 20° matched well with the (100) interplanar spacing of face-centered cubic graphitic carbon (sp2). X-ray photoelectron spectroscopy (XPS) was then used to investigate the surface chemical compositions. As shown in Fig. 1d, the survey spectra of MBCDs and BCDs-1 display peaks located at 285.0, 400.0, and 532.6 eV that are attributed to C 1 s, O 1 s, and N 1 s, and the atomic ratio are 62.52:35.75:1.73 and 57.64:39.21:3.15. The high-resolution spectra of C 1 s in Fig. 1e shows that the C = O, C–N, and C–C bonds are located at 286.3, 285.1 and 284.8 eV, respectively. Furthermore, the FT-IR spectrum of the as-prepared MBCDs and BCDs-1 display characteristic peaks at 1072, 1406, 1622, 2967, and 3336 cm-1, corresponding to C–O–C, C = C, C = O, C-H, and O–H stretching vibrations, respectively, which are highly consistent with the XPS analyses (Fig. 1f). The above data shows the close surface chemical compositions of the two samples. As shown in Fig. 1g and h, under 340 nm and 330 nm ultraviolet excitation, both MBCDs and BCDs-1 emitted blue light, with peaks at λ = 438 nm and λ = 435 nm, respectively. And they all presented a fluorescence emission dependence on the excitation light of different wavelengths. The corresponding emission gradually redshifted as the excitation light wavelength increased from 300 to 600 nm, indicating similar fluorescence characteristics. To systematically investigate all the as-prepared groups, we characterized BCDs-2 and BCDs-3 via the same techniques (detailed in Additional file 1: Fig. S1–S8). The results were highly consistent with the samples mentioned above.

### Biodistribution of MBCDs in mice organs and chronic organ toxicity

**Fig. 2:**
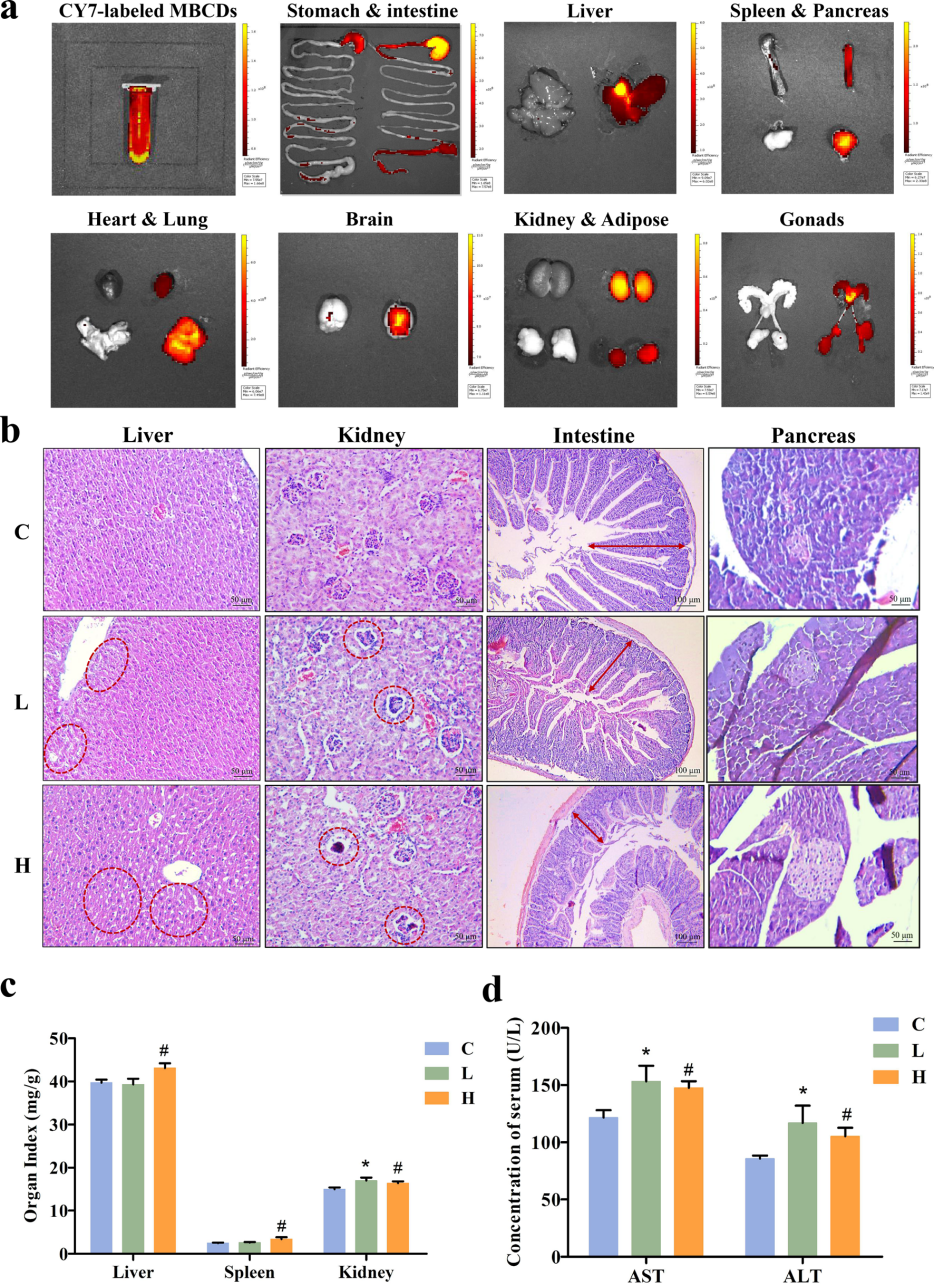
Biodistribution of the MBCDs in mouse organs, and organ damage caused by chronic oral administration. **a** Relative fluorescence intensity of major organs of mice injected with rhodamine CY7-labeled MBCDs via tail vein (n = 3). **b** H&E staining. **c** Organ indexes for mouse.** d** The levels of AST and ALT in serum. *p < 0.05 for L vs. C group, #*p* < 0.05 for H vs. C group. Results are the mean ± SE (n = 8)

To detect the biodistribution of MBCDs in the organs, we injected the mice with CY7-labeled MBCDs into the tail veins. As shown in Fig. 2a, we observed apparent fluorescent signals in the stomach, intestine, liver, spleen, pancreas, lungs, heart, brain, kidney, adipose, and gonads of the experimental mice. These signals confirmed that MBCDs circulated into the mice organs, especially the liver and pancreas, which are closely related to glucose metabolism.

The livers of mice exposed to 5 and 25 mg/kg of MBCDs for 16 weeks were paraffin-embedded for sectioning and staining. H&E staining is a common histopathological analysis technique. H&E results showed dose-dependent hepatocyte cord rupture, hepatic steatosis, glomerular atrophy, and intestinal villus shorten induced by MBCDs (Fig. 2b). The organs of mice were weighted to calculate the organ indexes (organ weight/body weight, mg/g). The Fig. 2c showed a significant increase in organ indexes in the experimental groups, especially in the liver (F: 3.67, *p*: 0.043). Moreover, the levels of AST and ALT (AST, F: 3.634, *p*: 0.0441; ALT, F: 4.524, p: 0.0232) in serum were significantly higher in MBCDs-treated groups (Fig. 2d), indicating MBCDs caused liver injury in mice.

### Chronic MBCDs intake induce glucose and insulin intolerance

**Fig. 3:**
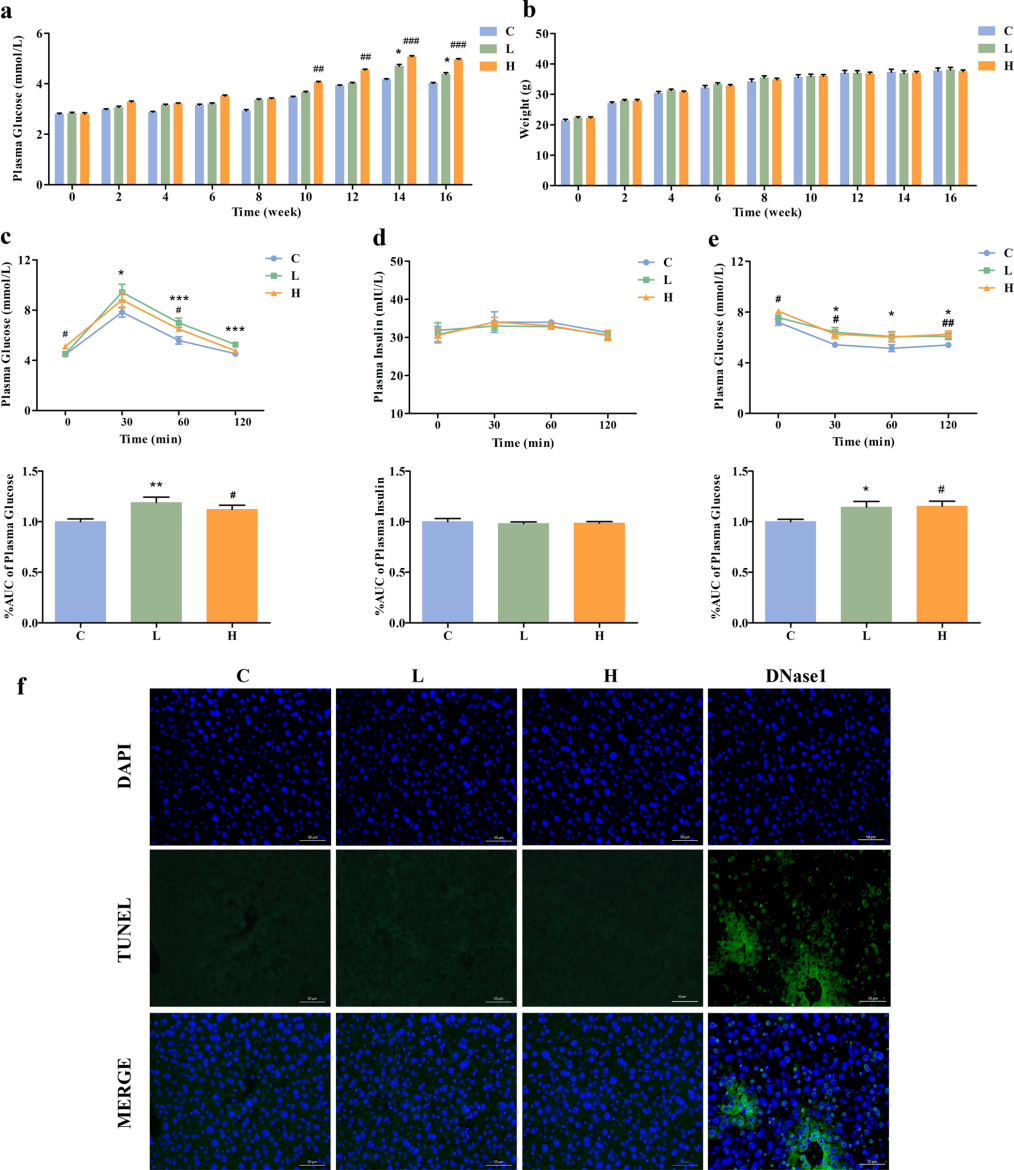
The oral administration of MBCDs disrupted glucose homeostasis in mice. **a** The blood glucose levels of mice tail veins. **b** Body weight. **c** Time course of changes and area under the curve (AUC) of blood glucose levels during the OGTT. **d** Time course of changes and AUC in blood insulin levels during the OGTT. **e** Time course of changes and AUC in blood glucose levels during the ITT. *p < 0.05 for L vs. C group, **p < 0.01 for L vs. C group, ***p < 0.001 for L vs. C group, #*p* < 0.05 for H vs. C group, ##*p* < 0.01 for H vs.C group, ###*p* < 0.001 for H vs. C group. Results are the mean ± SE (n = 8). **f** TUNEL assay apoptosis detection (FTIC) in liver cells. TUNEL (green) and nuclear (blue) constraining. TUNEL and nuclear staining are presented overlaid. DNase I was used as a TUNEL-positive control

After 10 weeks of daily oral MBCDs, high fasting blood glucose levels were observed in the experimental groups (F: 6.502, *p*: 0.0063) (Fig. 3a), while there were no significant differences in food intake and body weight with group C (Fig. 3b and Additional file 1: Fig. S9). This result indicates that the hyperglycemia induced by MBCDs was not caused by food intake. At week 14, an oral glucose tolerance test (OGTT) was used to determine the effects of MBCDs on glucose homeostasis. Notably, both MBCDs-treated groups developed obvious glucose intolerance (F: 5.285, *p*: 0.0138) (Fig. 3c). However, as shown in Fig. 3d, there were no significant effects on serum insulin concentration among the three groups within 120 min (F: 0.2312, *p*: 0.7956). At week 15, an insulin tolerance test (ITT) was performed after the mice were fasted for 6 h to examine insulin sensitivity. As shown in Fig. 3e, the ITT results reveal insulin intolerance was significant in both the 5 and 25 mg/kg groups after chronic MBCDs exposure (F: 3.666, *p*: 0.0431). TUNEL results showed that there was no cell apoptosis in the mice livers (Fig. 3f). These results prove that MBCDs caused glucose metabolism disorders but had no effect on liver insulin secretion and hepatic cell apoptosis, which was most likely caused by IR.

### RNA-seq revealed that chronic MBCDs intake induced oxidative stress in mice

**Fig. 4:**
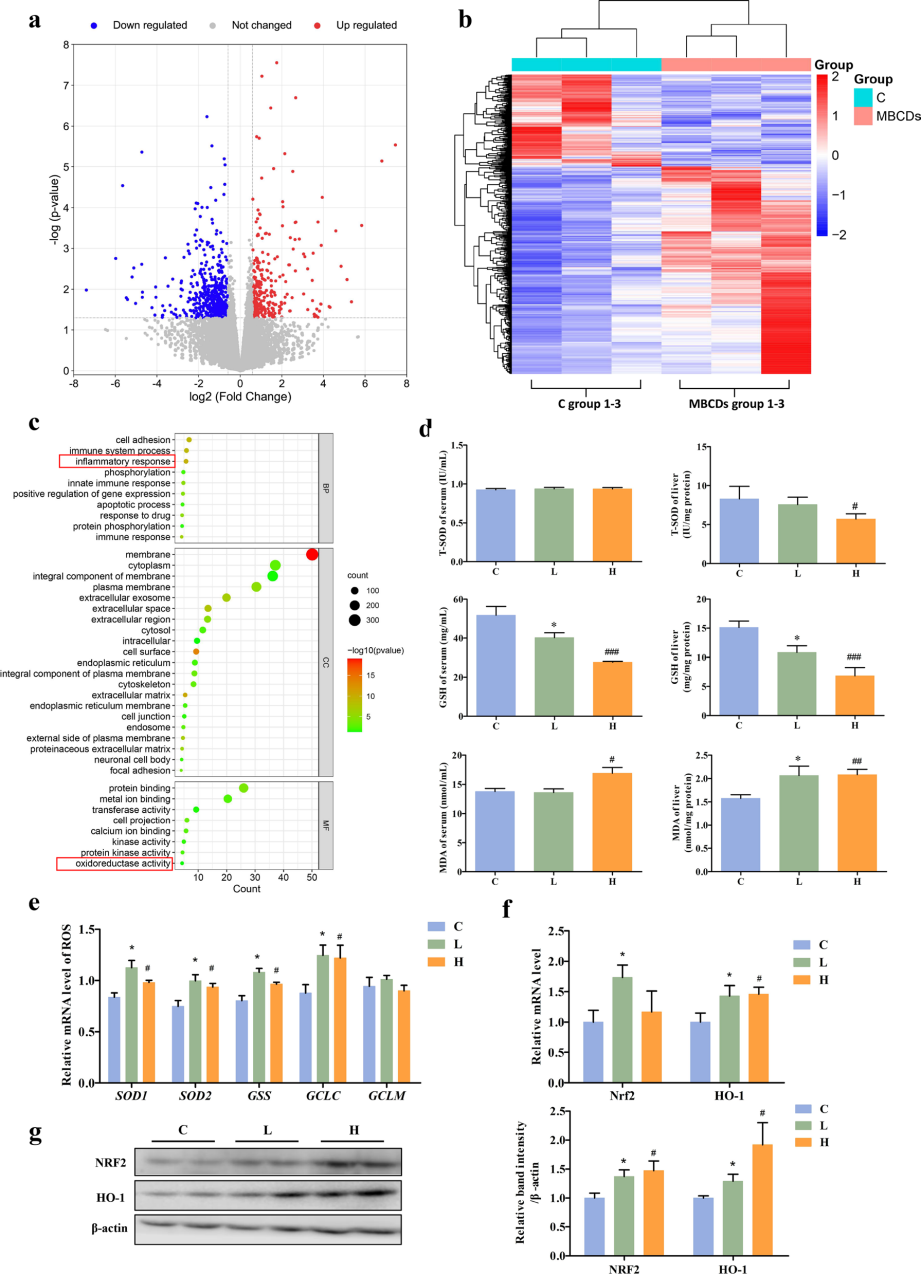
MBCDs induced mice liver oxidative stress. **a** Volcano plot of differential gene expression. **b** Cluster map of differential gene expression. **c** Top 30 GO terms. (n = 3). **d** The activities of total SOD (T-SOD) and glutathione (GSH) in the serum and the liver. The levels of malondialdehyde (MDA) in the serum and the liver. **e**, **f** Oxidative stress-related genes were validated by RT–qPCR. **g** Protein expression of Nrf2/HO-1 pathway. *p < 0.05 for L vs. C group, #*p* < 0.05 for H vs. C group, ##*p* < 0.01 for H vs.C group, ###*p* < 0.001 for H vs. C group. Results are the mean ± SE (n = 8)

To gain insight into the effect of MBCDs on the global gene expression of mice, transcriptomic analyses of mouse liver exposed to 25 mg/kg of MBCDs for 16 weeks were performed using RNA-seq. Notably, the mRNA abundance of 1126 genes was significantly changed in MBCD-treated mice, of which 494 were up-regulated and 632 were down-regulated (Figs. 4a and b). Functional annotation analysis of these differential genes was performed using the Annotation, Visualization and Integrated Discovery, (DAVID, https://david.ncifcrf.gov/home.jsp). All differential genes were enriched in 325 Gene Ontology (GO) terms and 53 Kyoto Encyclopedia of Genes and Genomes (KEGG) pathways (Fig. 4c, Additional file 1: Fig. S10 and Fig. S12). These results show that MBCDs affected the processes of oxidation–reduction (GO:0055114, GO:0016491, GO:0,016705), inflammatory response (GO:0006954, GO:0043123, GO:0051092, GO:0050729 GO:0043122, and GO:0043408), and glucose metabolism (GO:0061179, GO:0051055, GO:0006112, GO:0006629, GO:0061179, GO:0009311, mmu04151, and mmu040680).

Overproduction of intracellular ROS is an essential feature of oxidative stress; therefore, the detection of ROS-related biological indicators reflects the intracellular oxidative stress status. The results show that MBCDs significantly reduced T-SOD activity in the H group livers and GSH levels in the serum and livers of two groups (serum, F: 18.61, *p*: < 0.0001; liver, F: 10.93, *p*: 0.0006) (Fig. 4d), suggesting that MBCDs induced oxidative stress in mice. Furthermore, gene expression levels of superoxide dismutase 1 (SOD1) (4.593, *p*: 0.0221), superoxide dismutase 2 (SOD2) (F: 5.883, *p*: 0.0094), glutathione synthetase (GSS) (F: 9.174, *p*: 0.0014) and glutamate-cysteine ligase catalytic subunit (GCLC) (F: 3.902, *p*: 0.0362) were increased significantly (Fig. 4e). On this basis, the gene and protein expression levels of NRF2 and HO-1 in the mice livers were detected by western blot. In our work, the NRF2 gene and protein (gene, F: 2.244, *p*: 0.1308; protein, F: 3.846, *p*: 0.0377) were overexpressed in the experimental group, and HO-1 (gene, F: 3.111, *p*: 0.0656; protein, F: 4.129, *p*: 0.0307) showed the same trend (Fig. 4f and g). Compared with the C group, NRF2 and HO-1 were overexpressed in both treatment groups. All these results confirm that MBCDs induced oxidative stress in the mice.

### MBCDs-induced IR through ROS-induced inflammatory response

**Fig. 5:**
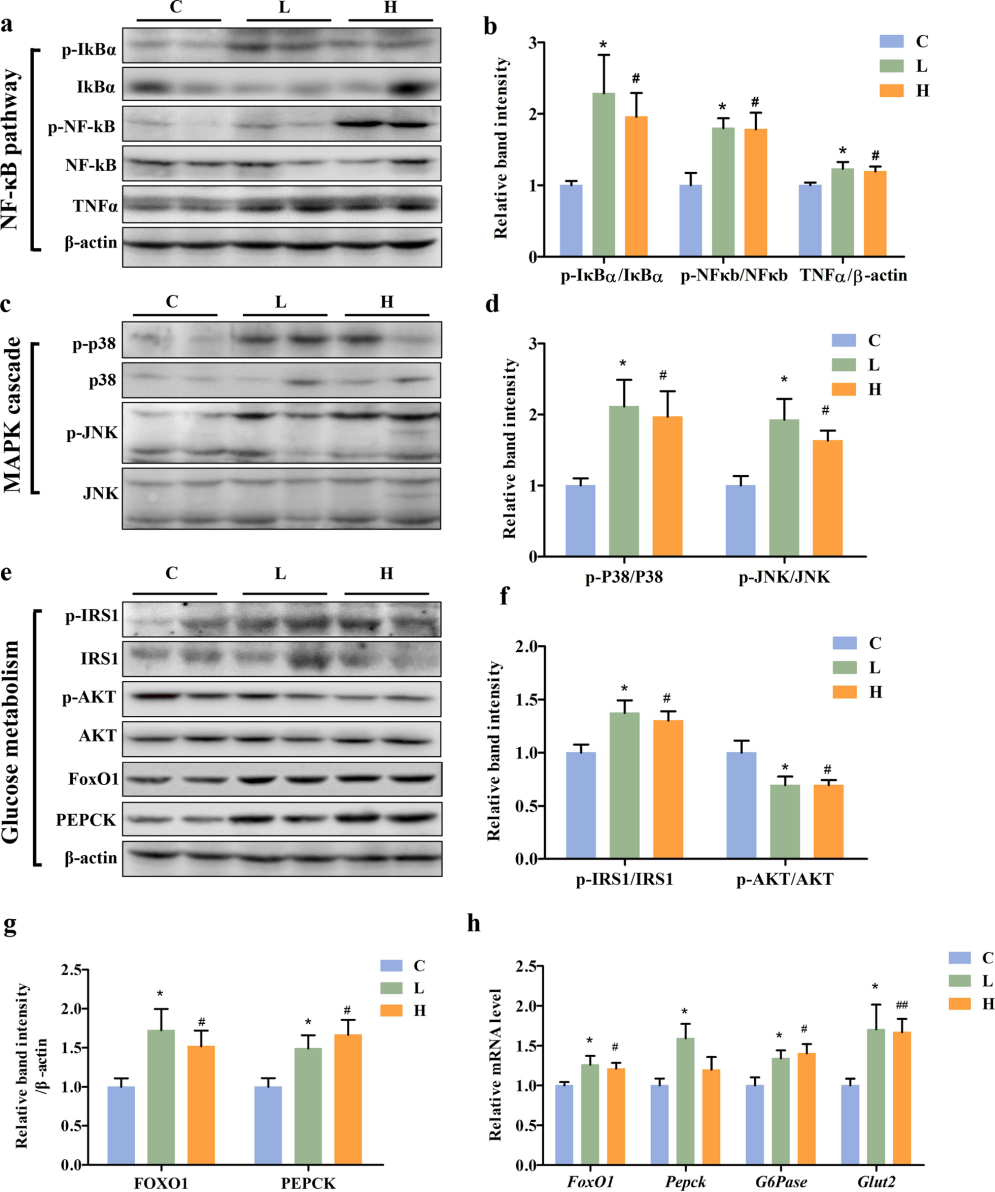
MBCDs-induced oxidative stress activated NF-κB and MAPK pathway, leading to IR and gluconeogenesis. **a**, **b** Western analyses of NF-κB pathway in liver of mice at 16 weeks after oral administration of MBCDs with different concentrations. **c**, **d** Western analyses of MAPK pathway. **e**–**g** Western analyses of glucose metabolism-related proteins. **h** Gluconeogenesis-related genes were validated by RT–qPCR. *p < 0.05 for L vs. C group, #*p* < 0.05 for H vs. C group, ##*p* < 0.01 for H vs. C group. Results are the mean ± SE (n = 8)

Western blot results show MBCDs significantly phosphorylated p65-NF-κB and IκB, suggesting that MBCDs induced an inflammatory response via the NF-κB/IκB pathway (p-NF-κB, F: 3.846, *p*: 0.0377; p-IκB, F: 3.795, *p*: 0.0392) (Fig. 5a and b). Furthermore, the protein expression of tumor necrosis factor alpha (TNF-α) in mice livers was examined by western blot, and the results show it was enhanced by the MBCDs. As an inflammatory cytokine, TNF-α can activate the MAPK cascade to promote IR further. In our work, we observed an activated MAPK cascade, which was reflected in phosphorylated p38-MAPK and JNK (p-p38 MAPK, F: 3.937, *p*: 0.0353; p-JNK, F: 5.359, *p*: 0.011) (Fig. 5c and d). Based on this, we examined the expression of glucose metabolism-related genes or proteins, including phosphorylated IRS1(Ser307), Akt (Ser473), FoxO1, PEPCK and G6Pase. As is shown in Fig. 5e–h, the MBCDs induced the phosphorylation of IRS-1 and dephosphorylation of AKT (p-IRS-1, F: 4.253, *p*: 0.0248; p-AKT, F: 4.214, *p*: 0.0289), increased the gene and protein expressions of FoxO1 (gene, F: 3.028, *p*: 0.0699; protein, F: 4.214, *p*: 0.0289), PEPCK (gene, F: 4.03, *p*: 0.033; protein, F: 4.593, *p*: 0.0221), and G6Pase (gene, F: 4.17, *p*: 0.0298), and upregulated the relative transcript levels of glucose transporter 2 (GLUT2) (gene, F: 3.607, *p*: 0.045), which promotes the transport of glucose from the liver to the blood. Overall, oral MBCDs blocked insulin signaling and promoted gluconeogenesis, leading to a significant increase in blood glucose levels in mice.

### Res and NAC inhibited MBCDs-induced ROS overproduction and hyperglycemia in mice

**Fig. 6:**
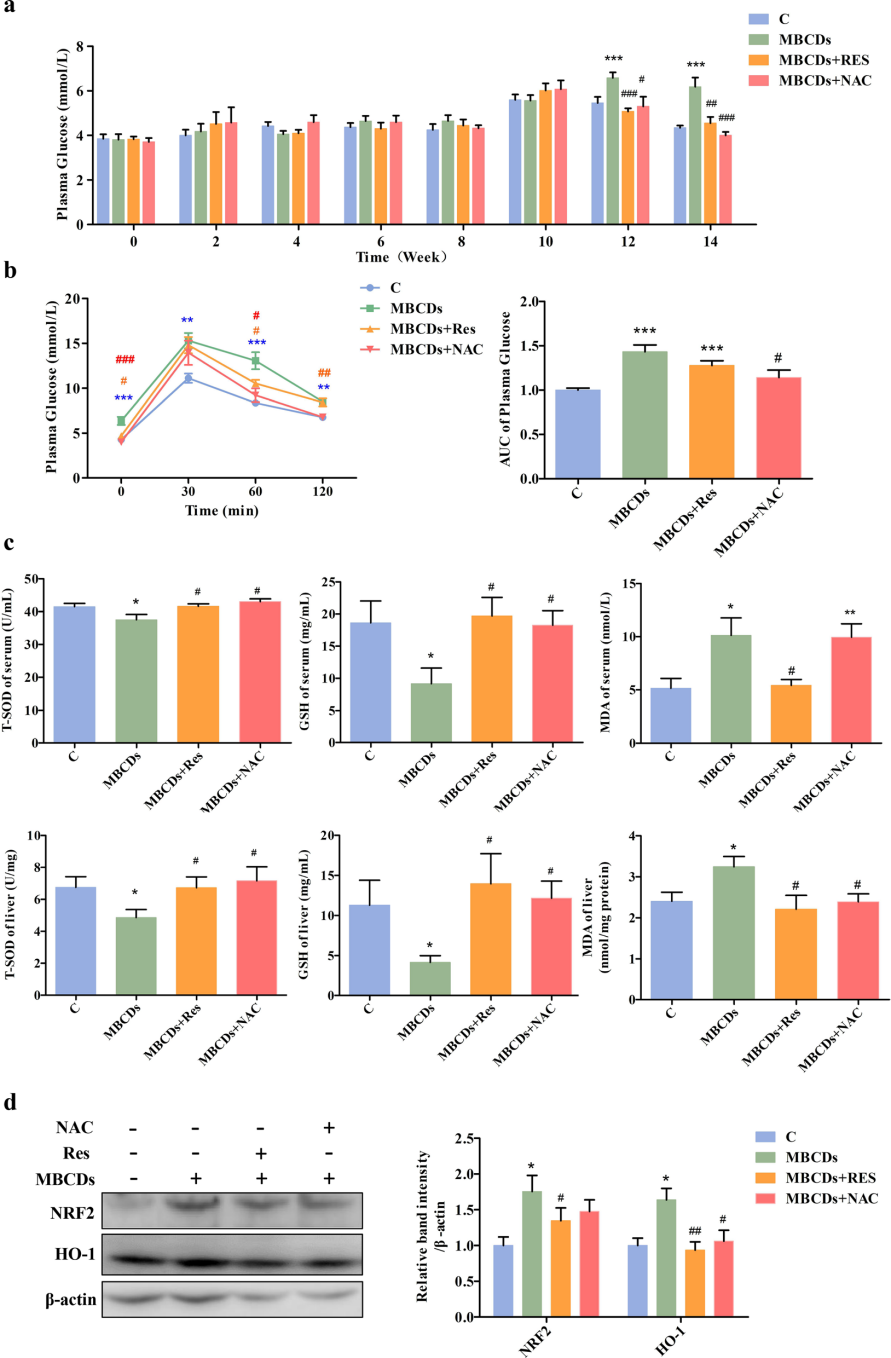
Res and NAC attenuated MBCDs-induced oxidative stress and glucose homeostasis imbalance. **a** The blood glucose levels of mice tail veins. **b** Time course of changes and area under the curve (AUC) of blood glucose levels during the OGTT. **c** The activities of total SOD (T-SOD) and glutathione (GSH) in the serum and the liver. The levels of malondialdehyde (MDA) in the serum and the liver. **d** Oxidative stress-related proteins were validated by western blot. *p < 0.05 for vs. C group, **p < 0.01 for vs. C group, ***p < 0.001 for vs. C group, #*p* < 0.05 for vs. MBCDs group, ##*p* < 0.01 for vs. MBCDs group, ###*p* < 0.001 for vs. MBCDs group. Results are the mean ± SE (n = 8)

To explore the mechanism of MBCDs-induced mouse glucose metabolism toxicity, we treated the mice with resveratrol (Res, 100 mg/kg) or N-Acetyl-L-Cysteine (NAC, 100 mg/kg), and administered 25 mg/kg MBCDs at the same time. The results show that Res and NAC effectively alleviated MBCDs-induced high levels of fasting blood glucose (F: 13.39, *p* < 0.0001) and glucose intolerance (F: 9.204, *p*:0.0002) (Fig. 6a and b). Moreover, a reduction of organ weight was observed in the Res and NAC groups (Additional file 1: Fig. S14.). We detected ROS-related biomarkers to verify the ROS inhibition of antioxidants. As shown in Fig. 6c T-SOD and GSH levels were significantly increased in serum and liver of antioxidant-treated mice, but MDA decreased rapidly (T-SOD serum, F: 5.247, *p*: 0.0053; T-SOD liver F: 2.761, *p*: 0.0607; GSH serum, F: 5.247, *p*: 0.0053; GSH liver F: 2.761, *p*: 0.0607; MDA serum F: 5.06; *p*:0.0063; MDA liver F: 3.75, p:0.0221). Meanwhile, the NRF2/HO-1 signaling was inhibited by Res and NAC (NRF2, F: 3.183, *p*: 0.0392; HO-1 gene, F: 5.796, *p*: 0.0033) (Fig. 6d).

### Res and NAC attenuated ROS-mediated MBCDs-induced insulin resistance

**Fig. 7:**
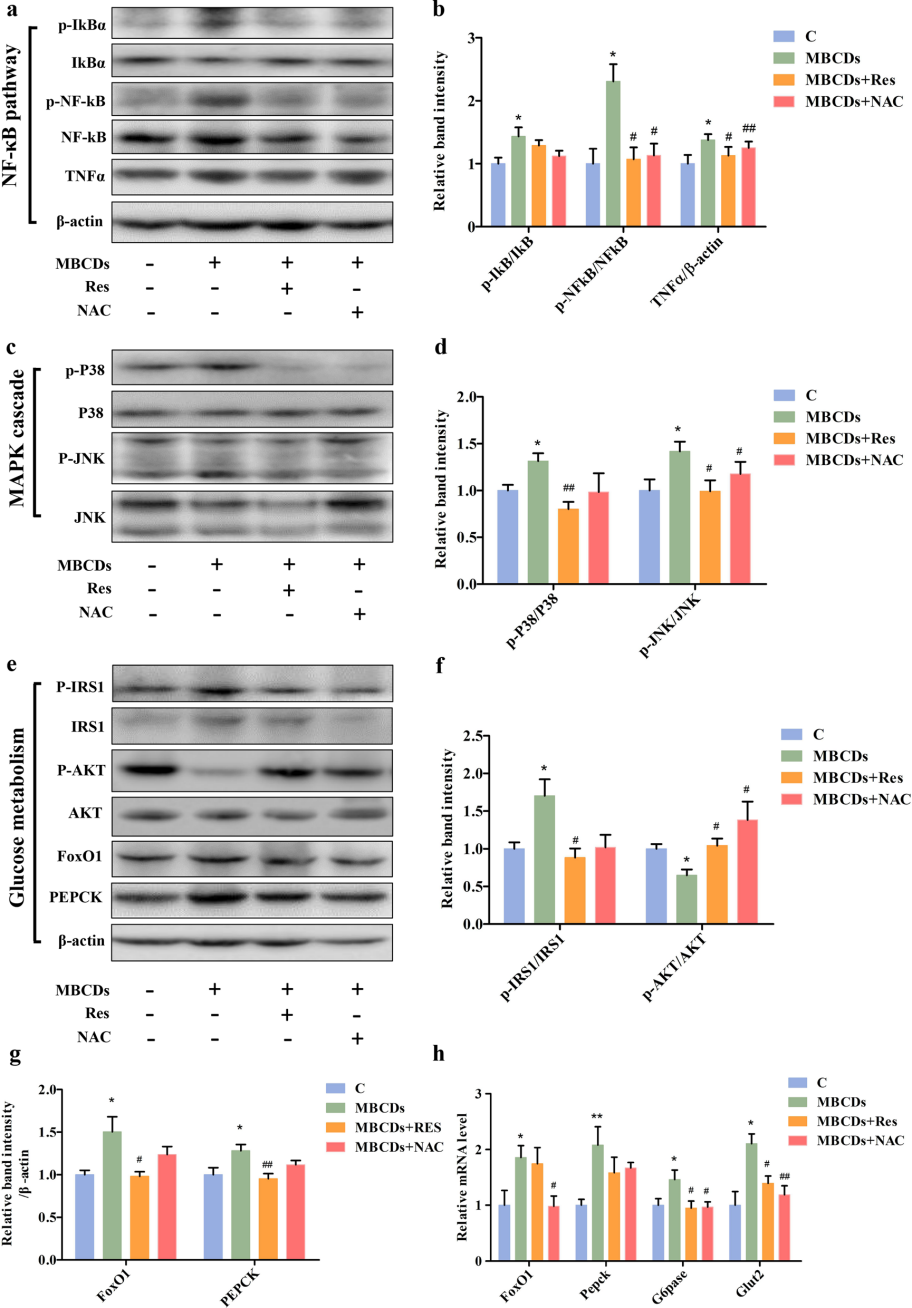
Res and NAC attenuated MBCD-activated NF-κB and MAPK pathway, and reduce IR and gluconeogenesis. **a**, **b** Western analyses of NF-κB pathway in liver of mice at 16 weeks after oral administration of MBCDs with different concentrations. **c**, **d** Western analyses of MAPK pathway. **e**–**g** Western analyses of glucose metabolism-related proteins. **h** Gluconeogenesis-related genes were validated by RT–qPCR. *p < 0.05 for vs. C group, **p < 0.01 for vs. C group, #*p* < 0.05 for vs. MBCDs group, ##*p* < 0.01 for vs. MBCDs group. Results are the mean ± SE (n = 8)

In the reverse experiment, antioxidant-mediated inhibition of ROS generation attenuated the MBCDs-induced inflammatory response, as evidenced by inhibition of phosphorylated p65-NF-κB and IκB and decreased TNFα (p-IκB, F: 3.325, *p*: 0.0399; p-NF-κB, F: 7.658, *p*: 0.0007; TNFα, F: 3.056, *p*: 0.0447) (Fig. 7a and b). Besides, we observed a suppressed MAPK cascade, which was reflected in the inhibition of phosphorylated p38-MAPK and JNK (p-p38 MAPK, F: 3.211, *p*: 0.0381; p-JNK, F: 3.191, *p*: 0.0388) (Fig. 7c and d). Furthermore, we detected the expression of glucose metabolism-related genes or proteins. In Fig. 7e–h, we found that Res and NAC attenuated MBCDs-induced phosphorylation of IRS-1 and dephosphorylation of AKT (p-IRS-1, F: 5.718, *p*: 0.0035; p-AKT, F: 4.663, *p*: 0.0091), indicating that MBCDs-induced IR was alleviated. Meanwhile, the expressions of FoxO1 (gene, F: 3.833, *p*: 0.0204; protein, F: 5.322, *p*: 0.005), PEPCK (gene, F: 3.893, *p*: 0.0192; protein, F: 4.801, *p*: 0.008), and G6Pase (gene, F: 3.658, *p*: 0.0242), and GLUT2 (gene, F: 9.402, *p*: 0.0012) were also decreased by Res and NAC. Above all, Res and NAC attenuated ROS-mediated MBCDs-induced inflammatory response and MAPK cascade further alleviate IR and gluconeogenesis.

## Discussion

The nanostructure contamination of food may occur along the food supply chain during the preparation, production, packaging, storage, or distribution [30–32]. Food-borne CDs produced during the normal food cooking process, and their size is closely related to the processing temperature [2]. Recently, the public has become more aware of the potential harm of food-borne CDs. CDs can significantly decrease cell viability, induce cell apoptosis, destroy the cell cycle, and cause metabolic stress [24]. However, few studies have focused on the health hazards of long-term intake of food-borne CDs, especially related to glucose metabolism. Poor dietary habits are the main cause of diabetes, and nanoparticles in food have been proven to induce hyperglycemia symptoms. Our results show that beer-borne CDs play a key role in diet-induced glucose homeostasis disorder that was previously unknown. Therefore, we emphasized the importance of revaluating food-borne CDs for food safety in our study.

A representative strategy was carried out to brew beer by using the malting barley as the main ingredients, then we extracted the CDs to explore how the MBCDs impaired mouse blood glucose metabolism. Moreover, various BCDs were extracted from different brands (Snow, Tsingtao, and Budweiser) of commercial beer we labelled BCDs-1, BCDs-2, and BCDs-3, respectively, to verify their similarity with MBCDs. The TEM and AFM images show the MBCDs have spherical shapes with narrow size distributions, and are close in size and morphology to BCDs. Furthermore, we observed similar morphologies and sizes of BCDs-2 and BCDs-3 as is shown in Additional file 1: Fig. S1-S8. The XRD patterns show similar crystalline structures as all the as-prepared samples, with only one characterized peak of carbon. To further investigate the composition of CDs, we characterized the XPS and FT-IR systematically. The MBCDs displayed peaks located at 285.0, 400.0, and 532.6 eV in the XPS spectrums attributed to C 1 s, N 1 s, and O 1 s, which was very similar to the BCDs. Furthermore, the FT-IR spectrums showed the same characteristic peaks (C–O–C, C = C, C = O, C-H, and O–H stretching vibrations) corresponding to all the prepared samples. Fluorescence is an important property of CDs, and we observed that the aqueous solution of all the as-prepared samples emitted blue color under UV light at 365 nm, which exhibited a similar fluorescence excitation dependence. The above results confirm the strong similarity between the MBCDs and BCDs of commercial beer.

In our study, CY7-labeled MBCDs were observed in vital metabolic organs of the mice 0.5 h after tail vein injection, suggesting MBCDs accumulated in mice organs. Moreover, oral administration of MBCDs for 16 weeks resulted in increased organ indexes and morphological damage of the livers, kidneys, and intestines, especially in the liver. ALT and AST are amino acid transferases and are markers for hepatic damage seen in hepatic diseases such as non-alcoholic fatty liver disease, liver cirrhosis, and T2DM [33]. The AST and ALT showed significant increases, indicating long-term intake of MBCDs may induce hepatotoxicity. Therefore, the potential endocrine toxicity of MBCDs needs further research.

Toxicological doses in animal models are generally related to body weight, and a correction factor (K_m_), estimated by dividing the average body weight (kg) of species to its body surface area (m^2^), can be used to estimate the human equivalent dose (HED) as: HED (mg / kg) = Animal dose (mg / kg) × K_m_ ratio [34]. Anroop et al. [35] reported that the Km ratio of mice-to-humans was 0.081. Although the daily intake of CDs from commercial beer is not yet clear, we calculated the extraction ratio of CDs in beer was approximately 0.0047% w/v. In this study, impaired glucose tolerance was observed in mice treated with MBCDs for 16 weeks at oral doses of 5 mg/kg and 25 mg/kg, corresponding to 0.405 mg/kg and 0.025 mg/kg daily intake in humans, respectively, which means that a 75 kg adult would consume 640 mL and 3 200 mL of beer per day. Sixteen weeks is approximately a fifth of a mouse’s lifespan, or 15 years in humans, suggesting that long-term, low dose CDs in beer may negatively affect glucose metabolism. Diabetes occurs as type I and type II with different pathogeneses. Type I diabetes mellitus (T1DM) is a metabolic syndrome characterized by insulin deficiency due to autoimmune destruction or dysfunction of islet beta-cells [36]. T2DM s is a pathological state in which cells cannot efficiently respond to insulin stimulation and is the result of insulin resistance due to impaired insulin signaling pathways [36]. In IR, pancreatic islets have normal insulin secretion but reduced glucose uptake and utilization [36]. The World Health Organization uses the oral glucose tolerance test (OGTT) and insulin tolerance test (ITT) to diagnose diabetes types [37]. In our study, the results of OGTT and ITT showed intolerance of glucose and decreased insulin sensitivity in MBCDs-treated mice, indicating the primary mechanism of MBCDs-induced hyperglycemia was IR. In addition, altered insulin secretion and apoptosis of hepatocytes were not observed, ruling out the underutilization of insulin by hepatocytes.

The liver is the classical insulin target organ and plays a key role in control of hepatic glucose homeostasis in diabetes [37]. In this study, we assessed the liver transcriptome to explore the mechanism of MBCDs-mediated imbalance of glucose homeostasis. The results showed that MBCDs activated the production of ROS. Overproduction of ROS mediates a number of oxidative damage diseases, including mitochondrial dysfunction, cellular aging, and apoptosis [38]. ROS also plays a critical role in the toxicological effects of nanoparticles (1–100 nm) on blood glucose metabolism [38–41]. Notably, the main genes enriched in ROS-related GO terms were belong to cytochrome P450 (CYP450) family, including cyp2b9, cyp21a1 and cyp4a12b (Additional file 1: Fig. S11a). And the mRNA expressions of cyp2b9, cyp21a1 and cyp4a12b were also significantly increased in the MBCDs-treated group (Additional file 1: Fig. S11b). Generally, ROS are mainly produced during the oxidative reaction of the mitochondrial respiratory chain and are by-products of normal cellular metabolism [42]. However, in certain conditions, such as the invasion of foreign substances, exogenous metabolic process in responses to the exposure to toxic compounds by the CYP450 enzymes can be another crucial important source of ROS [43]. CYP450 plays a role in scavenging foreign substances in cells, and can transform toxic metabolites into ROS, such as superoxide anion, hydrogen peroxide and hydroxyl radical which might cause injury of cells [44]. The monooxygenase system contributes significantly to the overall ROS formation in the cell [45]. When MBCDs enters the liver, hepatocytes activate the P450 gene and undergo (GO:0016491-oxidoreductase activity) and (GO:0016705-oxidoreductase activity, acting on paired donors, with incorporation or reduction of molecular oxygen) reaction to oxidize and expel foreign substances. Stimulated by MBCDs, the monooxygenase system releases superoxide anion and hydrogen peroxide through P450s in hepatocyte, and continuous production of ROS is an inevitable result of NADPH consumption by microsomal P450s. Total superoxide dismutase (T-SOD) and glutathione (GSH) are natural ROS scavengers in the body, which are consumed in removing ROS [46]. Malondialdehyde (MDA) is a metabolite of ROS and lipid response and is significantly increased in ROS overproduction. In our study, we identified MBCDs-induced ROS overproduction at multiple levels. The RT-qPCR results showed that MBCDs increased the expression of SOD1, SOD2, GSS, and GCLC at the mRNA levels, which encoded the endogenous antioxidants: T-SOD and GSH. Furthermore, T-SOD and GSH levels were significantly increased in serum and liver of antioxidant-treated mice, but MDA decreased rapidly, implying inhibition of MBCDs-induced overproduction of ROS. Therefore, in this study, MBCDs mainly generated ROS through P450s.

The nuclear regulator erythroid 2-related factor (Nrf2) is thought to be a cytoprotective factor that regulates the cellular oxidation reduction process and plays a vital role in in mammalian anti-oxidative stress. With overproduction of ROS, Nrf2 is released from Keap1 and binds to the antioxidant responsive elements (AREs), such as hemoxygenase-1 (HO-1) [47]. In our study, the expression of NRF2 and HO-1 was elevated in the livers of mice exposed to MBCDs compared with controls, suggesting that MBCDs-induced ROS overproduction activates NRF2/HO-1 signaling. Intriguingly, persuasive research reported that HO-1 deletion in mice evoked resistance to diet-induced IR and inflammation, dramatically reducing liver toxicity, in which the nuclear factor-κB (NF-κB) pathway plays a major role [48]. NF-κB is a family of transcription factors that plays a critical role in inflammation and is regulated by upstream phosphorylated IκB. Phosphorylation of IκB promotes translocation to the nucleus and phosphorylation of NF-κB p65 [49], producing the proinflammatory factor TNFα, which further promotes the inflammatory response. ROS can directly or indirectly (by stimulating high expression of HO-1) promote inflammatory responses. As shown in our RNA-seq results, MBCDs altered the inflammatory response (GO:0006954, GO:0043123, GO:0051092, GO:0050729, and GO:0043122). In our validation experiments, the activation of NF-κB signaling were observed in protein levels. That is, MBCDs-induced ROS overproduction in mouse hepatocytes promoted the NRF2/HO-1 and NF-κB signaling, and increased HO-1 also indirectly promoted NF-κB signaling to release proinflammatory factors and enhance cellular inflammation.

The inflammatory cytokine, TNFα, can directly phosphorylate insulin receptor substrates-1 (IRS-1) at Ser307 [50, 51], leading to the inhibition of insulin signaling, which in turn inhibits the phosphorylated protein of protein kinase B (AKT) at Ser473, resulting in IR. In addition, TNFα can also indirectly block insulin signaling by activating the mitogen-activated protein kinase (MAPK) cascade, leading to phosphorylation of p38 MAPK and c-Jun N-terminal kinase (JNK), which also contributes to phosphorylation of IRS1 (Ser307) [52]. Our RNA-seq results also showed significant changes in MAPK cascades (GO:0043410, GO:0043408, and GO:0043406). We examined hyperphosphorylation of p38 and JNK in protein levels, indicating MBCDs activated MAPK cascade through ROS-mediated inflammatory response. The enhanced phosphorylation of IRS-1 (Ser 307) and decreased phosphorylation of AKT (Ser 437), suggesting that ROS-mediated inflammation and MAPK cascade contribute to MBCDs-induced IR. Indeed, IR and gluconeogenesis naturally complement each other. Forkhead box O1 (FoxO1), a transcription factor regulating gluconeogenesis, is dephosphorylated in IR and controls hepatic glucose production by regulating phosphoenolpyruvate carboxykinase (PEPCK) and glucose-6-phosphatase (G6Pase) [53–55]. In our work, IR promoted the upregulation of inactivated FOXO1 expression, which further promoted PEPCK and G6Pase expression, then upregulated the relative transcriptional level of glucose transporter 2 (GLUT2), which is responsible for the transport of glucose from the liver to the blood, leading to aggravated gluconeogenesis and enhanced MBCDs-induced hyperglycemia in mice.

To further confirm ROS was a factor involved in the MBCDs-induced hyperglycemia, two major antioxidants, resveratrol (Res) and N-Acetyl-L-Cysteine (NAC), were used to alleviate hyperglycemia in mice [56, 57]. In this reverse study, Res and NAC effectively inhibited the overproduction of ROS and alleviated MBCDs-induced hyperglycemia and glucose homeostasis imbalance. Despite this contributing factor to the imbalance of glucose homeostasis, it was not the only one, which was reflected in the fact that Res alleviated, but did not completely eliminate MBCDs-induced glucose intolerance, indicating that additional mechanism remains to be explored. Moreover, decreased ROS inhibited the Nrf2/HO-1 pathway, further reduced the activation of the NF-κB pathway and MAPK cascade. The phosphorylation level of IRS1 (Ser307) was decreased by Res and NAC treatment, thereby reversing insulin signaling, suggesting that MBCDs-induced ROS-mediated IR was mediated by Nrf2/NF-κB/MAPK signaling. The expressions of FOXO1, PEPCK, G6Pase and GLUT2 were inhibited after antioxidant treatment, indicating gluconeogenesis was suppressed after IR reversal. These results provide evidence that MBCDs-induced ROS generation promoted IR and caused hyperglycemia.

**Fig. 8:**
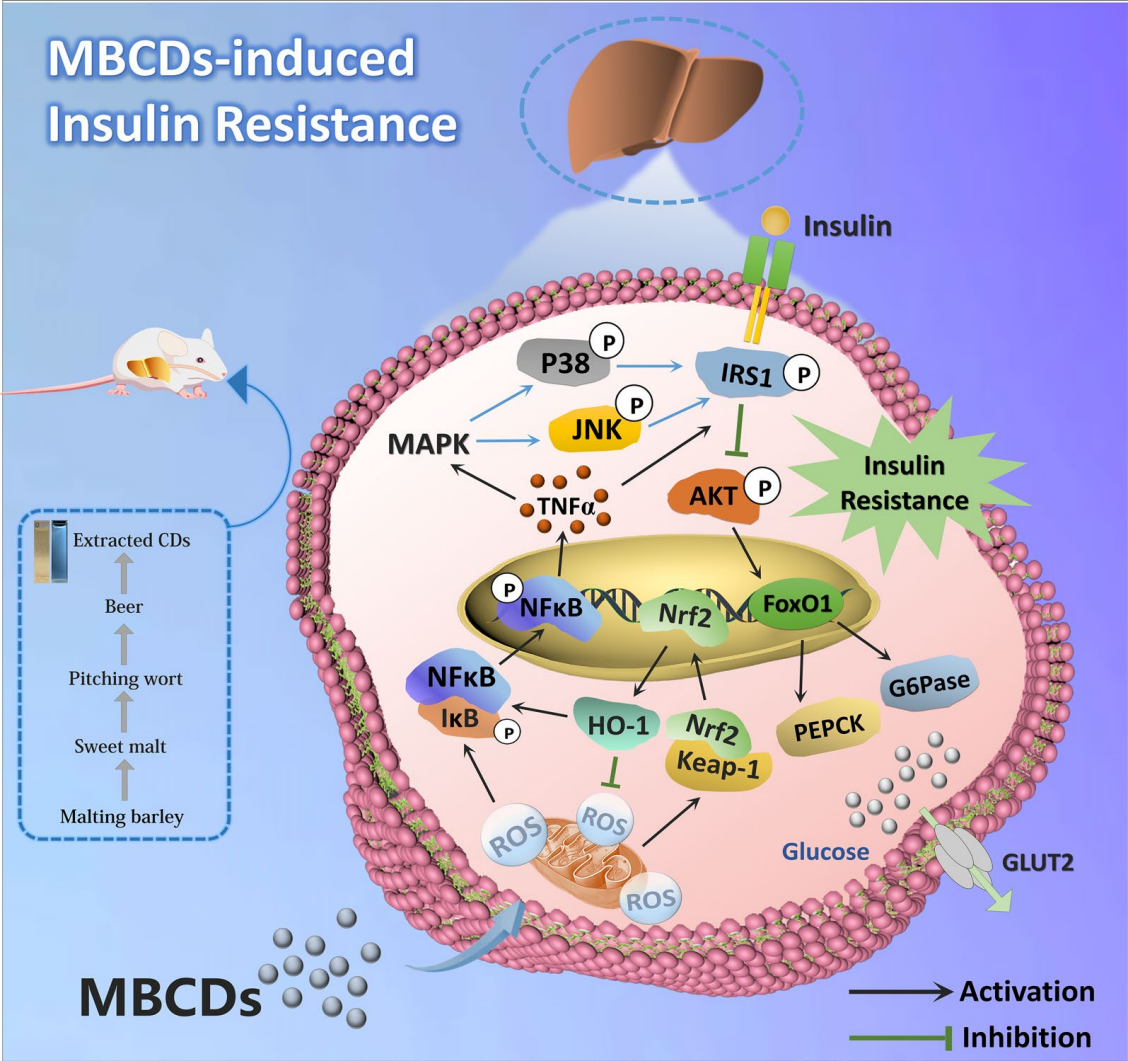
Schematic diagram of MBCDs intake effect on glucose metabolism in mice livers

As shown in Fig. 8, our results provide a potential mechanism by which MBCDs induced hyperglycemia in mice. MBCDs-induced ROS overproduction act as stimulating signaling molecules that activate the Nrf2/NF-κB/MAPK signaling, leading to IR and hyperglycemia. Our study provides evidence that chronic intake of CDs in beer may lead to an imbalance in glucose homeostasis, which can be ameliorated by inhibiting ROS production. This study provides an important reference for the scientific evaluation of the toxicological effects of foodborne CDs.

## Supplementary Information

**Additional file 1: Figure S1. **a) TEM and HRTEM images of the water-dispersed BCDs-2. b) TEM and HRTEM images of the water-dispersed BCDs-3. **Figure S2. **a) AFM images of the water-dispersed BCDs-2. b) AFM images of the water-dispersed BCDs-3. **Figure S3. **XRD spectrum of BCDs-2 and BCDs-3. **Figure S4. **FT-IR spectrum of BCDs-2 and BCDs-3. **Figure S5. **a) XPS spectrum and b) high revolution spectra of C 1s. **Figure S6. **High revolution spectra of N 1s of MBCDs, BCDs-1, BCDs-2 and BCDs-3. **Figure S7. **High revolution spectra of O 1s of MBCDs, BCDs-1, BCDs-2 and BCDs-3. **Figure S8. **a, b) Fluorescence spectrums of BCDs-2 and BCDs-3 excited at different wavelengths, and pictures of solution under daylight and ultraviolet conditions. **Figure S9. **a) Food intake. b) Water intake. **Figure S10. **GO terms related to glucose metabolism. **Figure S11.** a) Fold change of CYP450 genes. b) mRNA expressions of CYP450.** Figure S12. **MBCDs affected 53 Kyoto Encyclopedia of Genes and Genomes (KEGG) pathways in mice liver. **Figure S13. **Body weight of mice treated with MBCDs and MBCDs+antioxidants. **Figure S14. **Organ index and organ weight of mice treated with MBCDs and MBCDs+antioxidants. **Table S1.** Primer Sequence.

## Data Availability

The datasets used and/or analyzed during the current study are available from the corresponding author on reasonable request.
