# Supplementary material for: Malting barley carbon dots-mediated oxidative stress promotes insulin resistance in mice via NF-κB pathway and MAPK cascade
Source: J Nanobiotechnology. 2022 Jul 16;20:331. doi: 10.1186/s12951-022-01543-1 (PMC9288084; doi:10.1186/s12951-022-01543-1)
Supplement: Supplementary file 1 — Additional file 1: Figure S1. a) TEM and HRTEM images of the water-dispersed BCDs-2. b) TEM and HRTEM images of the water-dispersed BCDs-3. Figure S2. a) AFM images of the water-dispersed BCDs-2. b) AFM images of the water-dispersed BCDs-3. Figure S3. XRD spectrum of BCDs-2 and BCDs-3. Figure S4. FT-IR spectrum of BCDs-2 and BCDs-3. Figure S5. a) XPS spectrum and b) high revolution spectra of C 1s. Figure S6. High revolution spectra of N 1s of MBCDs, BCDs-1, BCDs-2 and BCDs-3. Figure S7. High revolution spectra of O 1s of MBCDs, BCDs-1, BCDs-2 and BCDs-3. Figure S8. a, b) Fluorescence spectrums of BCDs-2 and BCDs-3 excited at different wavelengths, and pictures of solution under daylight and ultraviolet conditions. Figure S9. a) Food intake. b) Water intake. Figure S10. GO terms related to glucose metabolism. Figure S11. a) Fold change of CYP450 genes. b) mRNA expressions of CYP450. Figure S12. MBCDs affected 53 Kyoto Encyclopedia of Genes and Genomes (KEGG) pathways in mice liver. Figure S13. Body weight of mice treated with MBCDs and MBCDs+antioxidants. Figure S14. Organ index and organ weight of mice treated with MBCDs and MBCDs+antioxidants. Table S1. Primer Sequence. [file 12951_2022_1543_MOESM1_ESM.pdf]

Supporting Information

Malting Barley Carbon Dots-mediated Oxidative Stress Promotes Insulin Resistance in Mice via NF-κB pathway and MAPK Cascade

*Boya Zhang[1,2], Lidong Yu[3], Ruijiao Zhu[1], Xiangjuan Wei[1], Xingpei Fan[1], Hailong Hu[4], Daqian Yang[1], Haining Du[1], Meimei Zhao[1], Li Li[1], Yuri Oh[5], Yuiie Feng[2], Ning Gu[1,2]**

[1] School of Life Science and Technology, Harbin Institute of Technology, 150001 Harbin, China.

[2] State Key Laboratory of Urban Water Resource and Environment, Harbin Institute of Techn ology, Harbin, 150006, China

[3] School of Physics, Harbin Institute of Technology, 150001 Harbin, China.

[4] Department of Medicine, Renal Electrolyte and Hypertension Division, Department of Genetics, Perelman School of Medicine, University of Pennsylvania, Philadelphia, PA, 19019, USA

[5] Faculty of Education, Wakayama University, Wakayama, Japan

*Corresponding authors.

E-mail: guning@hit.edu.cn (N. Gu)

Supplementary Figure 1

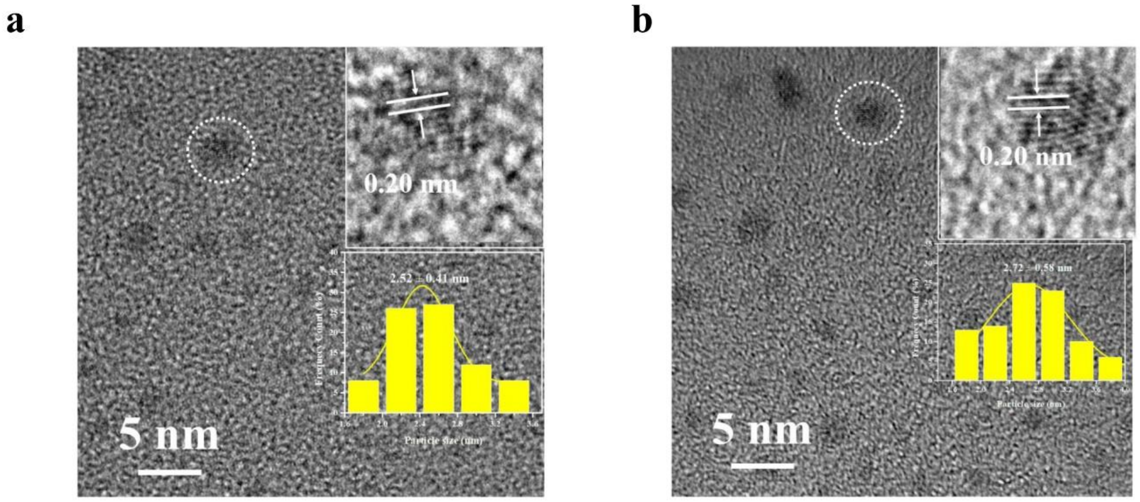

Supplementary Figure 1. a) TEM and HRTEM images of the water-dispersed BCDs-2. b) TEM and HRTEM images of the water-dispersed BCDs-3.

MBCDs and BCDs from self-brewing beer and different commercial beer (1: Snow, 2: Tsingtao, and 3: Budweiser) were extracted by a D101 macroporous resin column for comparative analysis. Transmission electron microscopy (TEM) was performed to characterize the nanostructure of the BCDs-2 and BCDs-3. As shown in **Supplementary Figure 1**, the TEM images display that BCDs-2 and BCDs-3 are well dispersed with narrow average diameters (2.52±0.41 nm and 2.72±0.58 nm) and exhibit substantial crystalline structures. Moreover, the corresponding high-resolution transmission electron microscopy (HRTEM) images show the same apparent interplanar spacing of 0.20 nm for the BCDs-2 and BCDs-3, which are highly consistent with the MBCDs.

Supplementary Figure 2

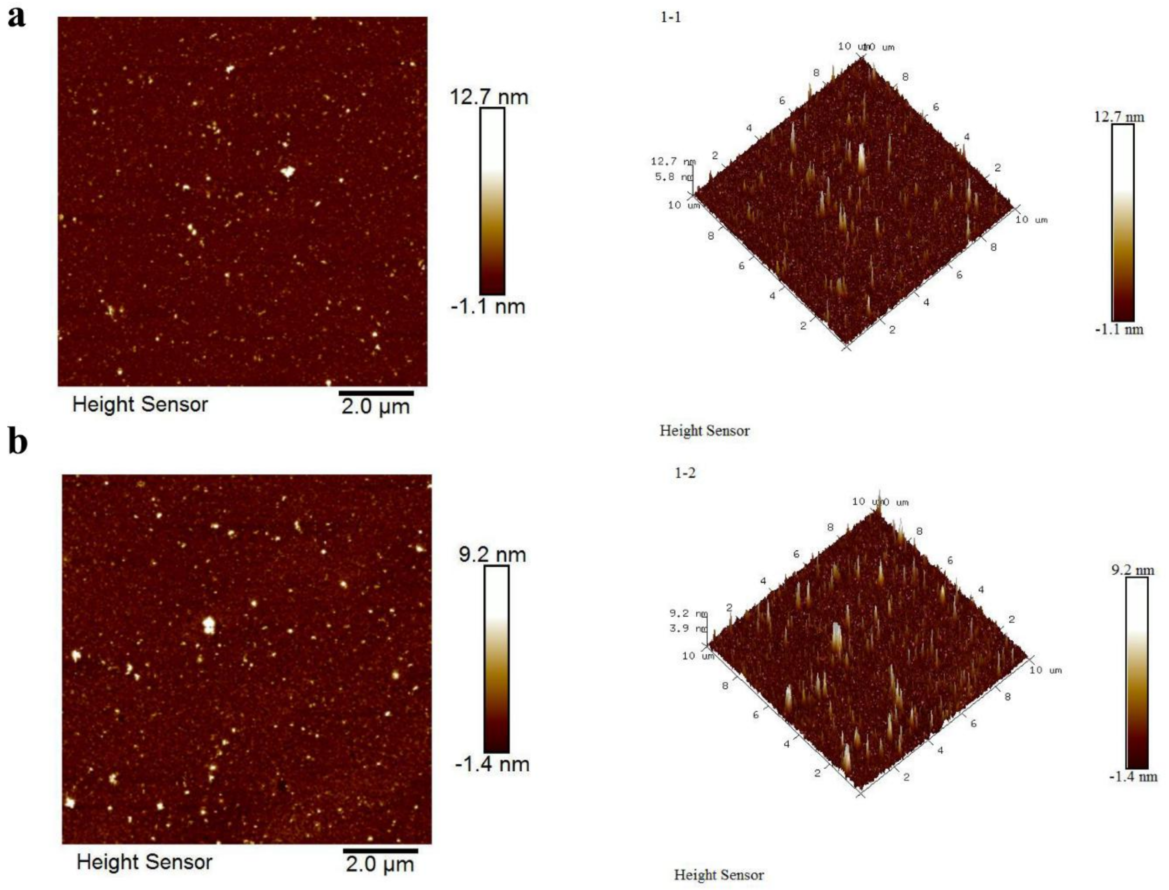

Supplementary Figure 2. a) AFM images of the water-dispersed BCDs-2. b) AFM images of the water-dispersed BCDs-3.

AFM technology was used to measure the three-dimensional morphological structure of BCDs-3 and BCDs-3, which exhibit the sharp morphology with an average height of 12.7 nm and 9.2 nm respectively, which are highly consistent with the MBCDs.

Supplementary Figure 3

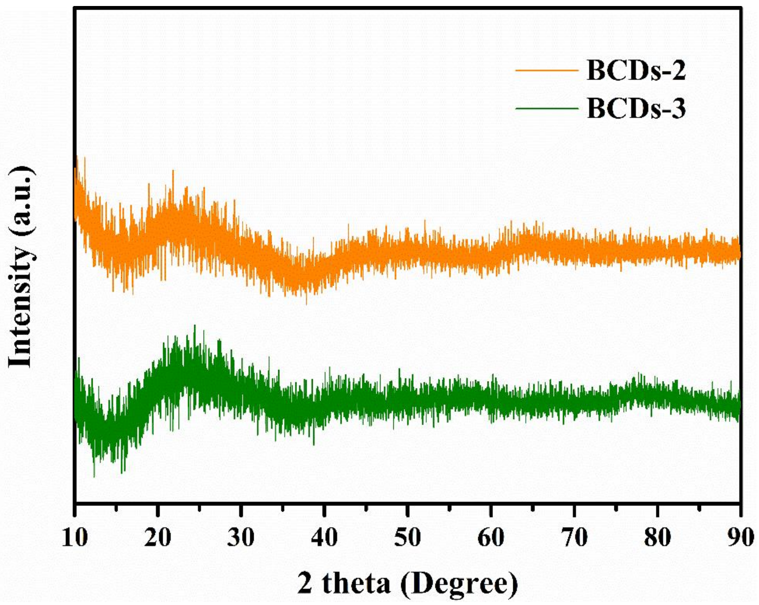

Supplementary Figure 3. XRD spectrum of BCDs-2 and BCDs-3.

The X-ray diffraction (XRD) pattern of BCDs-2 and BCDs-3 shows that the peak at 20° matched well with the (100) interplanar spacing of face-centered cubic graphitic carbon (sp2), which are highly consistent with the MBCDs.

Supplementary Figure 4

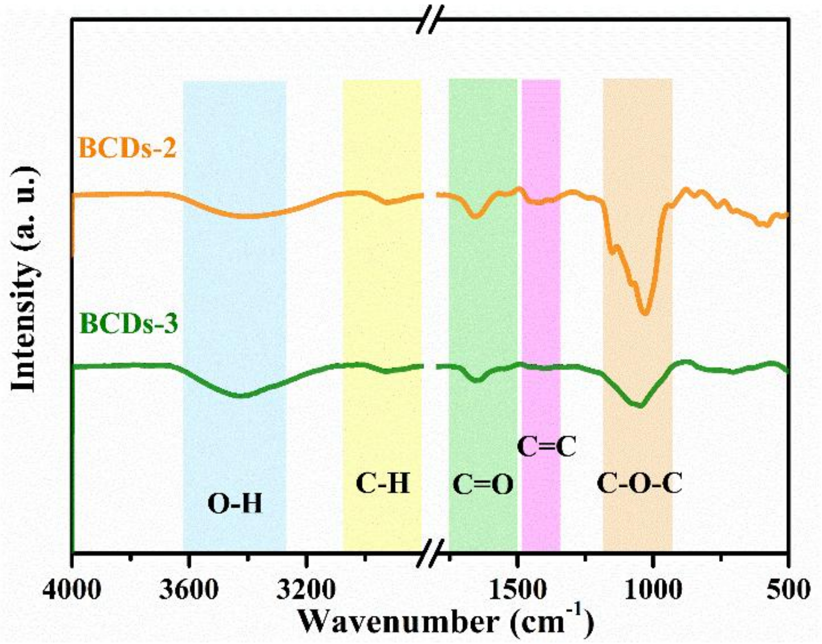

Supplementary Figure 4. FT-IR spectrum of BCDs-2 and BCDs-3.

Furthermore, the FT-IR spectrum of the as-prepared BCDs-2 and BCDs-3 display characteristic peaks at 1072, 1406, 1622, 2967, and 3336 cm-1, corresponding to C-O-C, C=C, C=O, C-H, and O-H stretching vibrations, respectively, which are highly consistent with the MBCDs.

Supplementary Figure 5

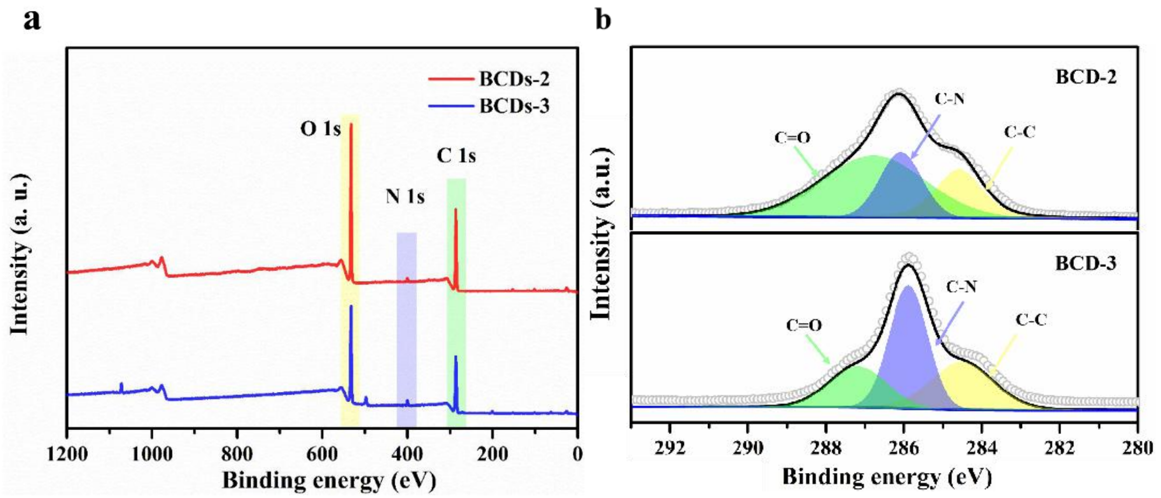

Supplementary Figure 5. a) XPS spectrum and b) high revolution spectra of C 1s.

X-ray photoelectron spectroscopy (XPS) was then used to investigate the surface chemical compositions. As shown in Figure 1d, the survey spectra of BCDs-2 and BCDs-3 display peaks located at 285.0, 400.0, and 532.6 eV that are attributed to C 1s, O 1s, and N 1s, and the atomic ratio are 58.95: 37.61: 3.43 and 57.49: 40.55: 1.96. The high-resolution spectra of C 1s in **Supplementary Figure 5b** shows that the C=O, C-N, and C-C bonds are located at 284.8, 285.1, and 286.3 eV, respectively.

Supplementary Figure 6

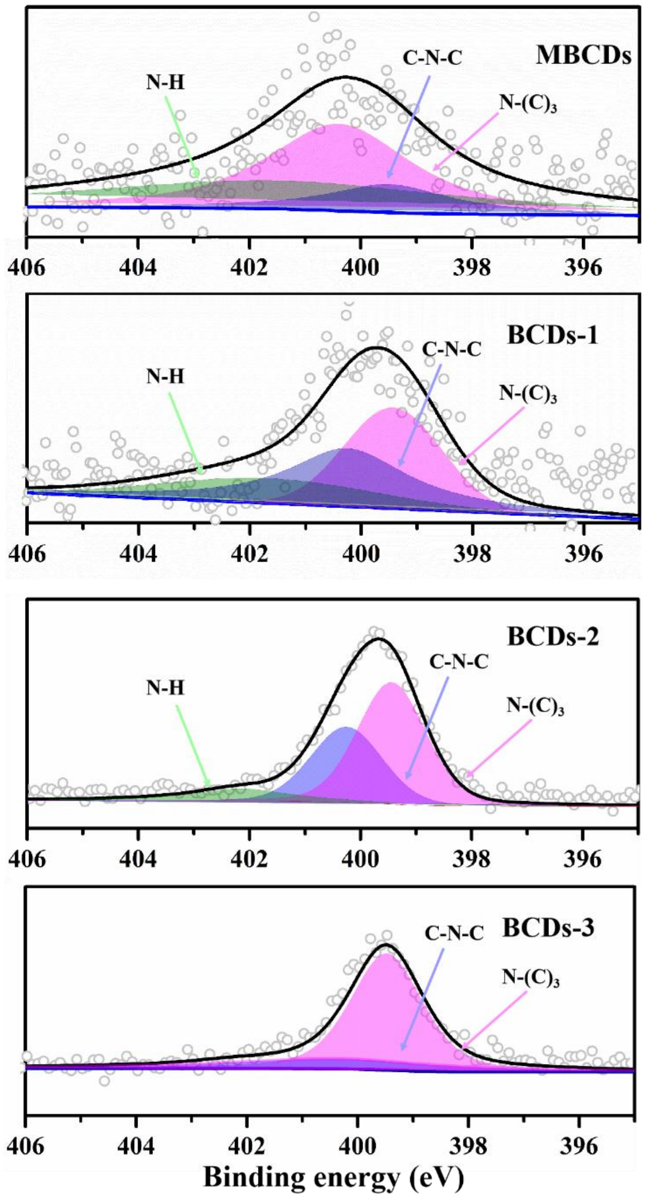

Supplementary Figure 6. High revolution spectra of N 1s of MBCDs, BCDs-1, BCDs-2 and BCDs-3. The high-resolution spectra of N 1s shows that the N-C3, C-N-C and N-H bonds are located at 399.5, 400.4, 401.5 eV, respectively.

Supplementary Figure 7

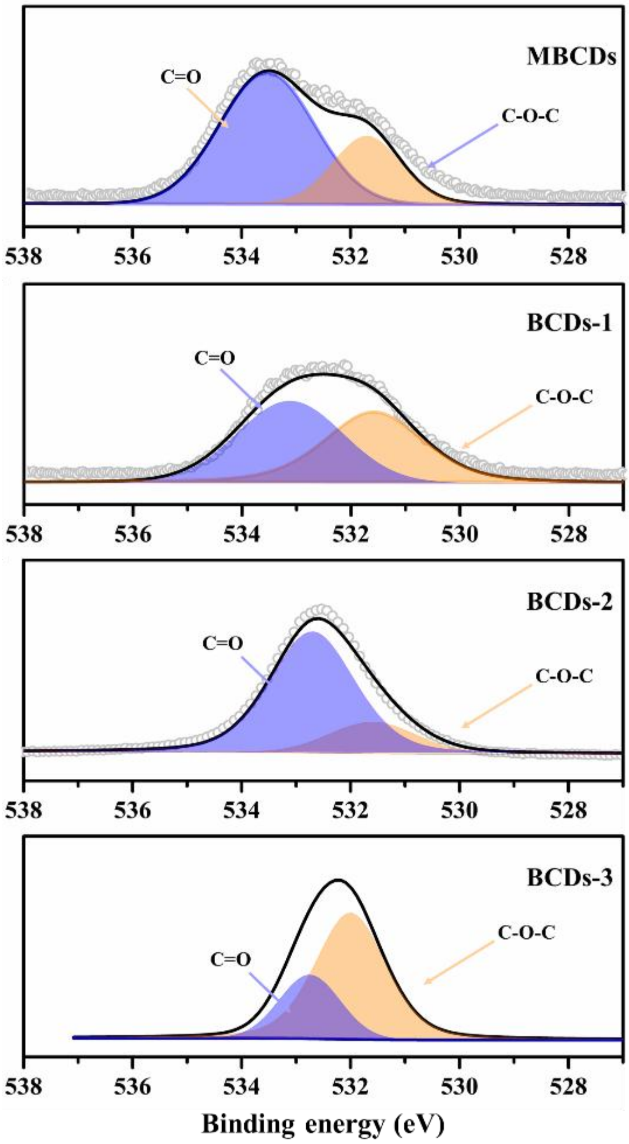

Supplementary Figure 7. High revolution spectra of O 1s of MBCDs, BCDs-1, BCDs-2 and BCDs-3. The high-resolution spectra of O 1s shows that the C-O-C/C-OH and C=O bonds are located at 399.5, 400.4, 401.5 eV, respectively.

Supplementary Figure 8

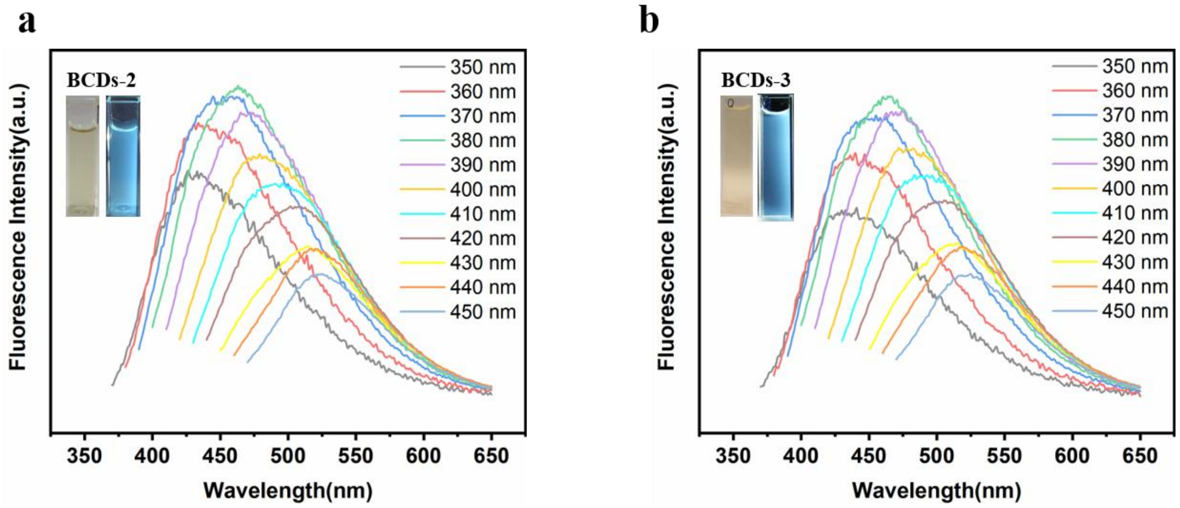

Supplementary Figure 8. a, b) Photoluminescence spectrums of BCDs-2 and BCDs-3 excited at different wavelengths, and pictures of solution under daylight and ultraviolet conditions.

As shown in **Supplementary Figure 8**, under 380 nm ultraviolet excitation, both BCDs-2 and BCDs-3 emitted blue light, with peaks at λ = 466 nm and λ = 463 nm, respectively. And they all presented a fluorescence emission dependence on the excitation light of different wavelengths. The corresponding emission gradually redshifted as the excitation light wavelength increased, indicating similar fluorescence characteristics.

Supplementary Figure 9

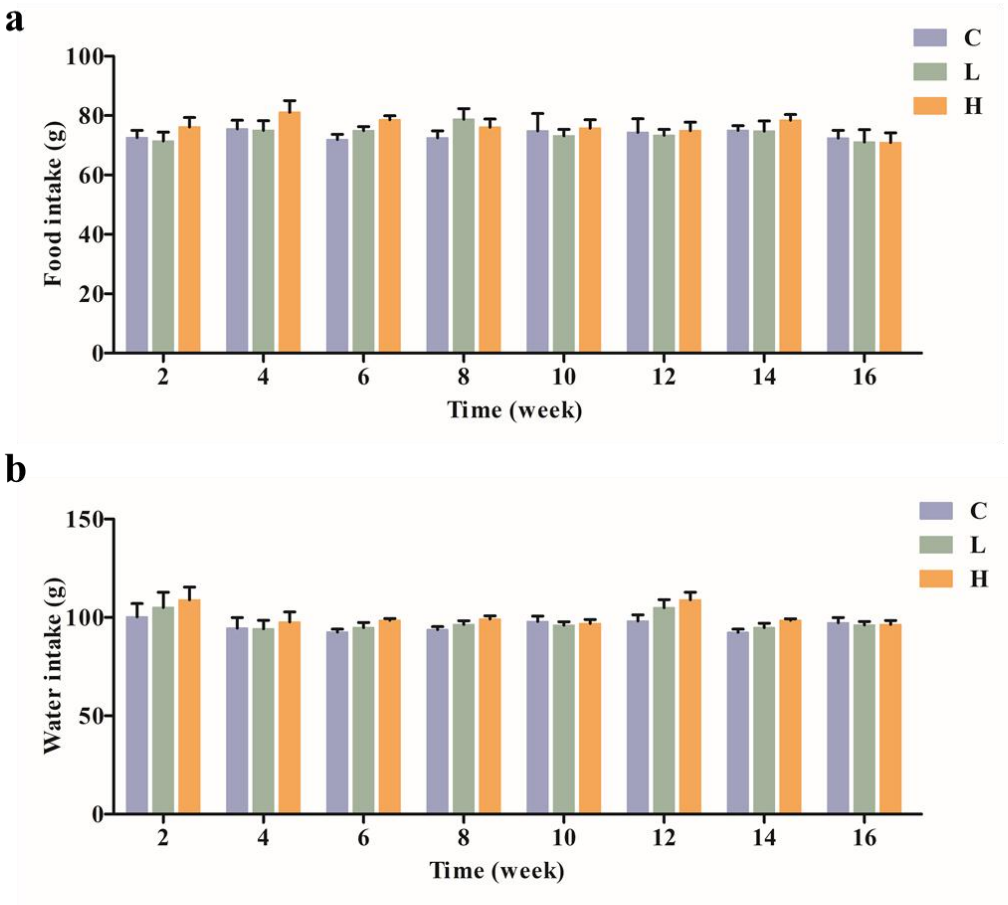

Supplementary Figure 9. a) Food intake. b) Water intake.

Since blood glucose is generally related to diet and water intake, we recorded these data of mice, and the results showed that the increase of fasting blood glucose in mice was independent of intake.

Supplementary Figure 10

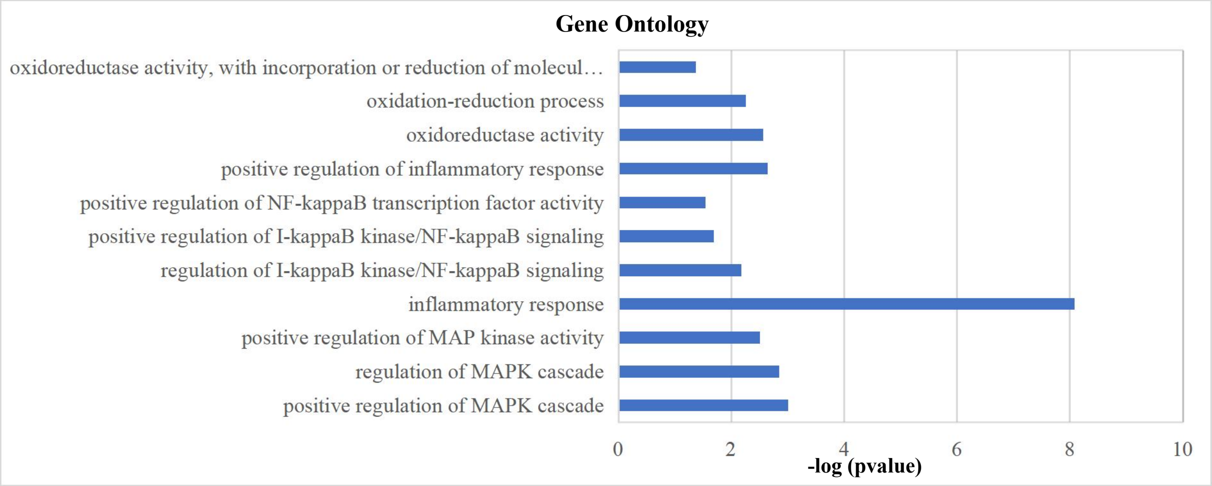

Supplementary Figure 10. GO terms related to glucose metabolism.

RNA-sequencing results revealed that 1126 genes significantly affected by oral administration of 25 mg/kg MBCDs were enriched in 325 gene Ontology (GO) terms and 53 Kyoto Encyclopedia of Genes and Genomes (KEGG) pathways. Among them, GO terms related to glucose metabolism are oxidative stress, inflammatory response and MAPK cascade respectively.

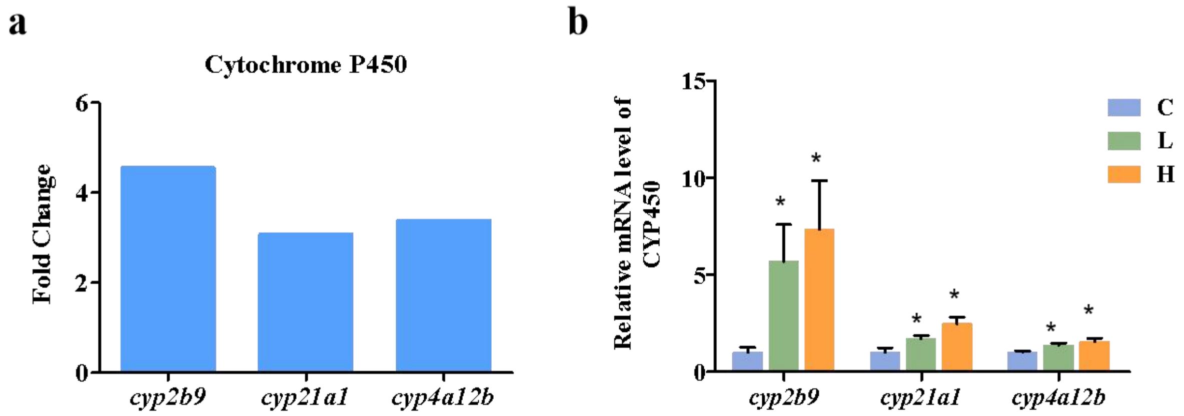

Supplementary Figure 11. a) Fold change of CYP450 genes. b) mRNA expressions of CYP450.

Supplementary Figure 12

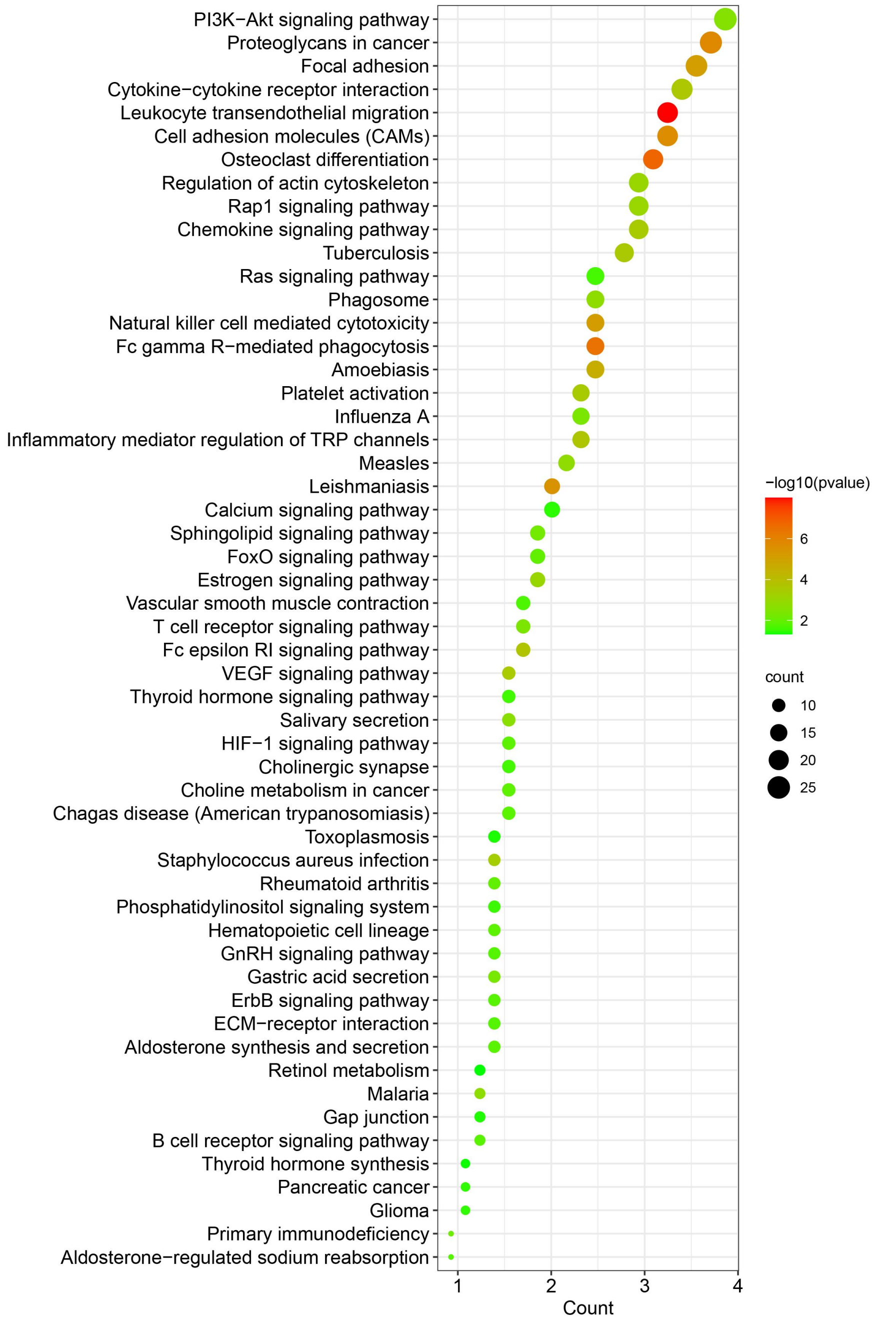

Supplementary Figure 12. MBCDs affected 53 Kyoto Encyclopedia of Genes and Genomes (KEGG) pathways in mice liver.

RNA-sequencing results revealed that 1126 genes significantly affected by oral administration of 25 mg/kg MBCDs were enriched in 53 Kyoto Encyclopedia of Genes and Genomes (KEGG) pathways. Among them, KEGG pathways related to glucose metabolism are PI3K-AKT signaling pathway and FoxO signaling pathway.

Supplementary Figure 13

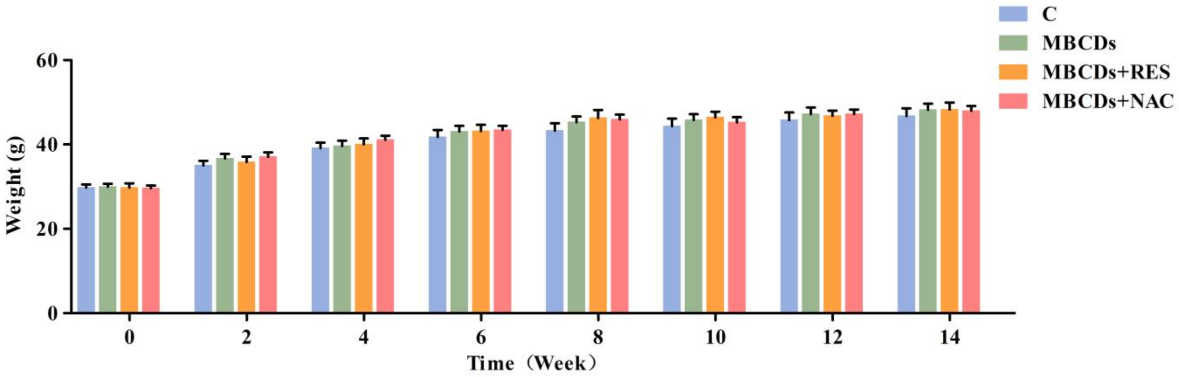

Supplementary Figure 13. Body weight of mice treated with MBCDs and MBCDs+antioxidants.

MBCDs and MBCDs+antioxidants treatment had no significant effect on the body weight of mice, suggesting the increase of fasting blood glucose in mice was independent of weight.

Supplementary Figure 14

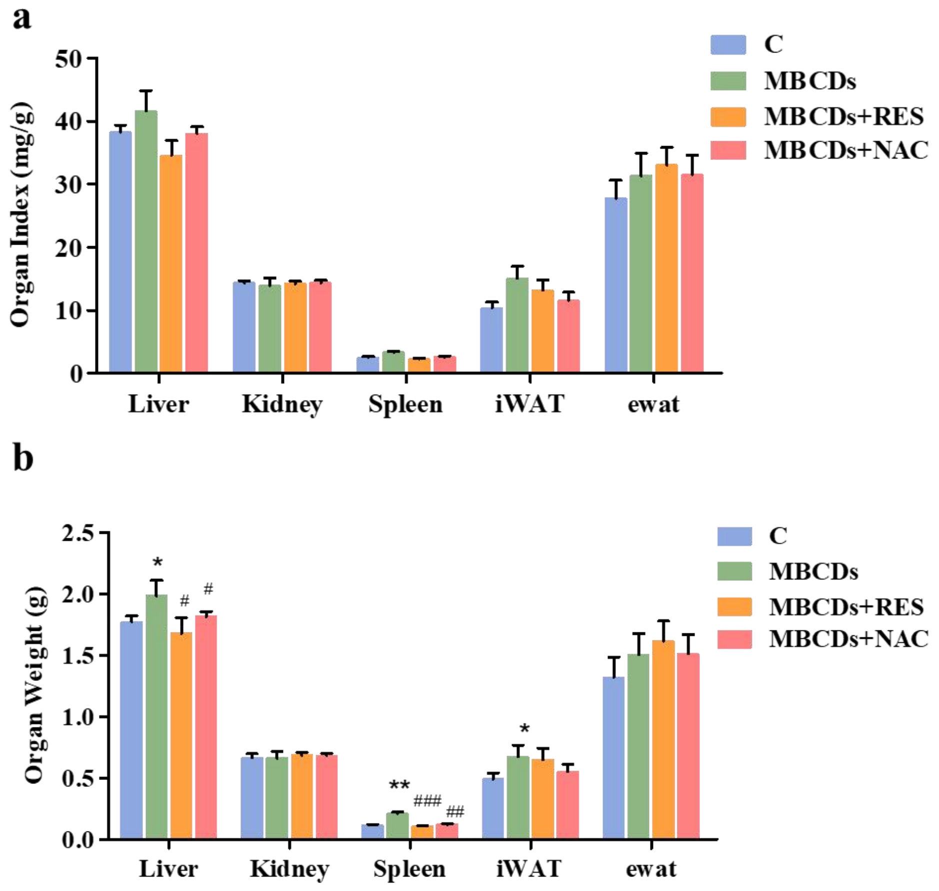

Supplementary Figure 14. Organ index and organ weight of mice treated with MBCDs and MBCDs+antioxidants.

MBCDs and MBCDs+antioxidants treatment had no significant effect on the organ index of mice, but had significant effect on organ weight.

Supplementary Table 1

primer	forward (5'-3')	reverse (5'-3')
36B4	GTAGTCAGTCTCCACAGACAAAGC	CCGTGTGAGGTCACAGTACC
GCLC	CTGCACATCTACCACGCAGT	GTCTCAAGAACATCGCCTCC
GCLM	CGGGAACCTGCTCAACTG	CCAAAACATCTGGAAACTCCC
GSS	GAAGCAGCTCGAAGAACTGG	AGCACTGGGTACTGGTGAGG
HO-1	CCCACCAAGTTCAAACAGCTC	AGGAAGGCGGTCTTAGCC
Nrf2	CAGCTACTCCCAGGTTGCCCACATT	GCCAAACTTGCTCCATGTCCTGCTCTAT
Nqo1	TGGCCGAACACAAGAAGCTGGAA	CCCCGTGGACACCCTGAAGAGAGT
SOD1	ACCATCCACTTCGAGCAGAA	AAAATGAGGTCCTGCACTGG
SOD2	AACTCAGGTCGCTCTTCAGC	GCTTGATAGCCTCCAGCAAC
FOXO1	ACGAGTGGATGGTGAAGAGC	ACGAGTGGATGGTGAAGAGC
PEPCK	TGAACTGACAGACTCGCCCT	GTCTTCCCACAGGCACTAGG
G6Pase	CGACTCGCTATCTCCAAGTGA	GGGCGTTGTCCAAACAGAAT
GLUT2	TCAGAAGACAAGATCACCGGA	GCTGGTGTGACTGTAAGTGGG
CYP21A1	CCTTGCCCCATCGTGCAACTA	TGGAGGCAGCAGAGTGAAGG
CYP4A12B	CAAGAAACTCTCTCGCTCAGCCCT	CAACGTGCTGTCCTTGTCTCCAAA
CYP2B9	TCCACCCTGGAGATCTTCCA	GCACACGGAGAAAAAGAGATTG
